# Review: Precision medicine and driver mutations: Computational methods, functional assays and conformational principles for interpreting cancer drivers

**DOI:** 10.1371/journal.pcbi.1006658

**Published:** 2019-03-28

**Authors:** Ruth Nussinov, Hyunbum Jang, Chung-Jung Tsai, Feixiong Cheng

**Affiliations:** 1 Computational Structural Biology Section, Basic Science Program, Frederick National Laboratory for Cancer Research, National Cancer Institute, Frederick, Maryland, United States of America; 2 Department of Human Molecular Genetics and Biochemistry, Sackler School of Medicine, Tel Aviv University, Tel Aviv, Israel; 3 Genomic Medicine Institute, Lerner Research Institute, Cleveland Clinic, Cleveland, Ohio, United States of America; 4 Department of Molecular Medicine, Cleveland Clinic Lerner College of Medicine, Case Western Reserve University, Cleveland, Ohio, United States of America; National Institutes of Health, UNITED STATES

## Abstract

At the root of the so-called precision medicine or precision oncology, which is our focus here, is the hypothesis that cancer treatment would be considerably better if therapies were guided by a tumor’s genomic alterations. This hypothesis has sparked major initiatives focusing on whole-genome and/or exome sequencing, creation of large databases, and developing tools for their statistical analyses—all aspiring to identify actionable alterations, and thus molecular targets, in a patient. At the center of the massive amount of collected sequence data is their interpretations that largely rest on statistical analysis and phenotypic observations. Statistics is vital, because it guides identification of cancer-driving alterations. However, statistics of mutations do not identify a change in protein conformation; therefore, it may not define sufficiently accurate actionable mutations, neglecting those that are rare. Among the many thematic overviews of precision oncology, this review innovates by further comprehensively including precision pharmacology, and within this framework, articulating its protein structural landscape and consequences to cellular signaling pathways. It provides the underlying physicochemical basis, thereby also opening the door to a broader community.

## Introduction

What exactly is “precision oncology,” and how can we, as a community, contribute to it? Despite its captivating terminology, the concept is not new; after all, it has long been known that to be effective, specific ailments necessitate tailored treatments [1]. However, its current genomic basis distinguishes it from its outmoded cellular renditions [2, 3]. The term “precision medicine”,or “precision oncology,” as in our case here, is commonly used when the treatment strategies involve targeted therapies that are based on personal data from next-generation sequencing [4–14]. That is, rather than select therapies based on the perceived type of cancer, for example, tissue or organ location, therapeutic selection is based on analysis of the individual’s genomic sequence and the specific identified mutational aberrations [15–18]. It is the mutation-guided therapeutics, rather than the traditional cancer type-dependence classification, such as that based on classical anatomy and histology, that has etched a new context into this terminology. This concept has compelled a paradigm shift. Now patients with *BRAF* V600E mutations would be prescribed the same drug regimen irrespective of their cancer type and location, for example, acute myeloid leukemia, breast cancer, or melanoma [19].

Precision oncology was not always based on the individual’s genomic sequence. Since its inception, exactly what precision oncology includes has been unclear. In 2015, Collins and Varmus proposed that blood-typing—based targeted therapies and immune therapy be included [20]. A 2017 analysis revealed that in the literature, “precision oncology” appears to have undergone an evolution [21]. Early on, therapies were disease and/or protein targeted, such as, for example, vascular endothelial growth factor (VEGF) inhibitors and Bcr-abl1 tyrosine kinase inhibitors (TKIs), bevacizumab (Avastin) and imatinib, respectively. Later, precision oncology treatments were referred to as selections of therapies based on analyses of biomarkers. Examples include crizotinib (Xalkori) in lung cancer with *EML4-ALK* rearrangements or adjuvant chemotherapy as in the Oncotype DX panel in breast cancer. The literature analysis observed that, only as of January 2016, precision oncology therapeutic selection appears to have been primarily based on next-generation sequencing data. As noted by Tsang and colleagues, various terms have been used to relate to precision genomic oncology, including simply precision oncology [22], genomics-driven oncology [23], genomic oncology, and personalized cancer medicine. All refer to high-throughput sequencing to inform clinical decisions at the point-of-care [24].

Even though the conceptual foundation of precision oncology is rational, thus stimulating broad enthusiasm, its current lack of demonstrated clear breakthroughs in clinical trials argues that in addition to more patient sequence data, critical components may be missing. Next-generation sequencing of patients with advanced cancers showed that less than 10% have actionable mutations [25, 26], and a randomized trial of precision medicine [27] did not observe improved outcomes with genome-based precision oncology. The biological complexity underlying target identification is challenging. The breakthrough of the next-generation sequencing delivered a new precision component: treatments might be tailored, not only to a certain illness but also to a specific person’s genetic make-up. This notion of prescribing “the right drug to the right person at the right time” [28–32] has stimulated considerable research efforts, which have been pushed to the fore by the Precision Medicine Initiative (also called *All of Us* Research Program). But to date, it seems to still fall short, and the magnitude of the task is daunting. The mutational landscape is highly heterogeneous and challenging to decipher. Data indicate that the least mutated cancers have on average 0.28 mutations per megabase, with most presenting 8.15 mutations per megabase [21, 33]. Whole-exome analysis of pancreatic cancer, which is considered only moderately mutated, indicates 2.64 mutations per megabase. Further disconcerting is the low consistency among mutated genes. For example, certain mutations are observed fairly rarely in pancreatic cancer and are observed in other tissues as well [34]. Additional factors tied to cancer and individual complexities cast long and formidable shadows as well.

Therefore, even though it is broadly believed that precision oncology can improve treatments and prognosis, and that precision data are essential for precision oncology, the consensus is that this may not be enough [35]. Current clinical results do not question the sequence-based hypothesis and strategies, but they do emphasize the need for considering their completeness [36]. The literature thrives with proposed additional clinical considerations, and vital statistical and network tools are also being developed [37, 38]. One powerful element that we believe is missing is the energy landscape; biomolecules are not static sculptures but interconvert between structures with varying energies [39]. Therefore, on its own, genomic sequence data may not provide the entire information to the oncologist in target selection. If the mutations are in protein coding regions, which is our focus here, then they translate to dynamic protein conformational ensembles and interactions, which in principle, we, as a community, can exploit to accomplish more accurate prognosis.

The low consistency among mutated genes in cancer argues that only translating sequence alterations to heterogeneous ensembles may not be sufficient, and new concepts should be brought to bear. One of these [40] is the notion that we should not only rely on classical categories and definitions of passenger mutations (appearing to confer no survival advantage) and driver actionable mutations (which propel cancer initiation and progression) [41–49]. Passenger mutations whose effects on their own appear insignificant may transform into driver mutations when acting in certain combinations. We dubbed such mutations latent drivers [49]. The low consistency of mutations among genes also argues that focusing on single proteins may be lacking. Additionally, mutations can take place during cancer evolution within the same or different tissues [50].

Cancer disrupts normal physiological tissue homeostasis due to loss of function or gain of function, which can take place in multiple ways [51–82]. Even though the underlying tenet of precision oncology largely rests on the notion that reversing one target will halt cancer, parallel signaling pathways leading to the same cellular outcome, compensatory mutations, and more are not overlooked. These are taken up through combinatorial drug regimens.

The excellent literature reviews and research papers in this area cover clinical and social benefits, as well as genomic sequence analysis, pattern identification, and approaches and/ or corroboration of target discovery. This latter category produces vital software tools [83–96]. Recently, functional advances were reviewed as well [97]. Below, following a background, we relate to precision oncology from a different standpoint. Our premise is the sensitivity of the free energy landscape to its environment [98–104]. We view actionable mutations within this framework, as well as their consequences for cell-specific signaling networks, and finally, we comment on how our community can help.

## Definition: “Protein space” and “protein conformation”

The theme of the paper is the change of population of states caused by various factors, including mutations. Within this framework, the general terms “protein space” and “protein conformation” refer to “energy states.” Protein space and conformations are discussed as having components as energy, conformation, and dynamics.

## Precision pharmacology

Is precision medicine, or its precision pharmacology abettor, a “hype” or is it real? And if it is, is it attainable? First off, the precision medicine initiative is not aimed at developing “personalized drugs,” which is infeasible. Instead, pharmacogenomics studies are carried out on groups of patients, and the drugs are tested in clinical trials, which inherently involve patients. Therefore, personalized drug development is not a priori for an individual; instead, the underlying premise is that it could be incorporated into cancer treatments fitting certain individuals, with the expectation that within that population, individual responses will vary. Because certain genetic variants in individuals can interfere with drug processing, the Food and Drug Administration (FDA) recommends genetic testing before giving a chemotherapy drug, as in the case of mercaptopurine (Purinethol) in acute lymphoblastic leukemia. Most drugs developed within this framework are in oncology. Still, only approximately 13% of the oncology drugs approved since Herceptin in 1998 have an FDA-approved personalized Companion Diagnostic, that is, laboratory analysis and clinical evaluation [105]; however, since 2016, 29 out of 30 with FDA-approved personalized companion diagnostics assays are in oncology. A major reason that most drugs that have been developed are in oncology is that the assays that are involved largely reflect single point mutations, as in the case of *KRAS* [106–131]. This makes the clinical result easier to interpret, as compared to more complex diseases involving multiple disease-associated genes.

Pharmacogenomics aims to develop effective and safe drugs for patients bearing specific genetic signatures. It integrates pharmacology and genomics, with the premise that a person’s response to a drug will depend on their genetic makeup. Whereas next-generation sequencing aims to identify driver mutations and molecular targets, pharmacogenomics aims to forecast how a person’s genetic makeup will affect their response to the drugs. Classically, drugs were developed for diseases with the assumption that a drug works similarly for all patients. By contrast, precision pharmacology countered the “one size fits all” notion, and because all physician treatments are personal, the personalized medicine concept has been renamed precision medicine [132–134].

**Fig 1:**
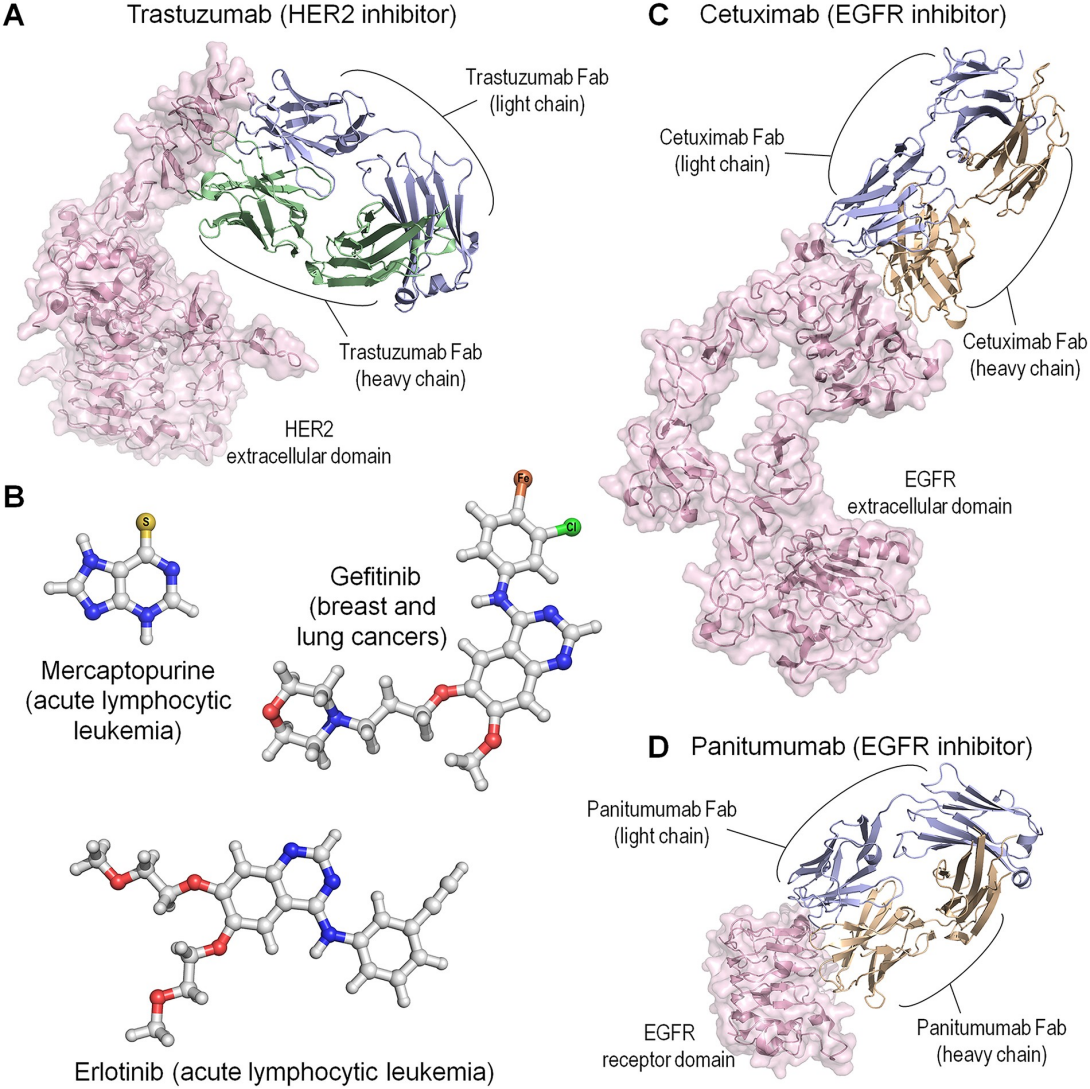
Examples of precision pharmacology. (A) Crystal structure of HER2 extracellular domain in complex with trastuzumab (Herceptin) Fab (PDB code: 1N8Z). Trastuzumab is a monoclonal antibody targeting HER2 receptor positive for metastatic breast cancer. (B) Molecular structures of mercaptopurine (Purinethol), gefitinib (Iressa), and erlotinib (Tarceva). Mercaptopurine is specifically used to treat acute lymphocytic leukemia. Gefitinib is an EGFR inhibitor used to treat breast, lung, and other cancers. Erlotinib is also an EGFR inhibitor used to treat NSCLC, pancreatic cancer, and several other types of cancer. In the structure, C, N, and O atoms are denoted as white, blue, and red spheres, respectively. Hydrogen atom is represented as an edge of stick. Other heavy atoms, S, Cl, and Fe atoms, are marked on the sphere. Molecular topologies with the coordinates are generated by using Avogadro software [412]. (C) Crystal structures of EGFR extracellular domain in complex with cetuximab (Erbitux) Fab (PDB code: 1YY9) and (D) EGFR receptor domain 3 in complex with panitumumab (Vecitibix) Fab (PDB code: 5SX4). Both cetuximab and panitumumab are monoclonal antibodies targeting EGFR. EGFR, epidermal growth factor receptor; Fab, antigen-binding fragment; HER2, human epidermal growth factor receptor 2; NSCLC, non-small cell lung cancer; PDB, protein data bank.

One example of precision pharmacology is the breast cancer drug trastuzumab (Herceptin) discussed above, which works only for tumors involving human epidermal growth factor receptor 2 (HER2)-overexpression and/or -amplification (Fig 1A). A second example is the chemotherapy drug mercaptopurine (Purinethol) against acute lymphoblastic leukemia (Fig 1B). Patients with a genetic variant that impedes processing of this drug can suffer from severe side effects. Chemotherapy drugs gefitinib (Iressa) and erlotinib (Tarceva) show improved outcome in patients with certain lung cancer genetic change. In contrast, chemotherapy drugs cetuximab (Erbitux) and panitumumab (Vecitibix) (Fig 1C and 1D) do not present good efficacy in 40% of colon cancer patients with certain genomic alteration. To be successful, pharmacogenomics data need not only be extensive but also go beyond one gene for one disease with one technology platform at a time. Precision pharmacology implies that rather than screening for compounds with broad action against a disease, genomic information should guide drug development for subgroups of patients with specific genetic profiles.

Data point to a need for enhancing the theory underlying the precision oncology framework. The elucidation of the human genome sequence [135] and identification of alterations that drive distinct cancers take place at an increasingly rapid pace, revealing additional genomic variants and more complex scenarios [132, 136]. Unlike the earlier imatinib and trastuzumab, the targeted therapies that were discovered recently are not as specific, suggesting drugs that might target such complexity are becoming available. This inspired the “actionable mutation” terminology, unraveling additional candidate targets [61, 90].

**Fig 2:**
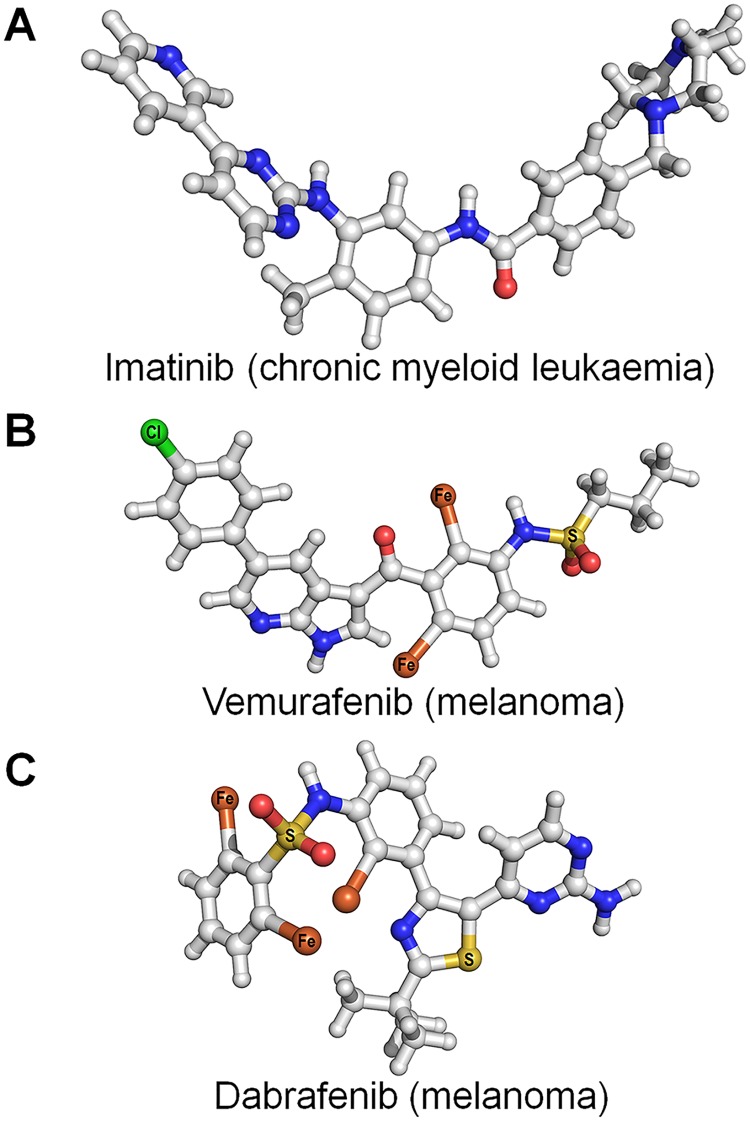
Examples of chemotherapy drugs. Molecular structures of (A) imatinib, (B) vemurafenib, and (C) dabrafenib. Imatinib is used for chronic myelogenous leukemia and acute lymphocytic leukemia. Both vemurafenib and dabrafenib are drugs for the treatment of melanoma targeting the B-Raf^V600E^ mutant. In the structure, C, N, and O atoms are denoted as white, blue, and red spheres, respectively. Hydrogen atom is represented as an edge of stick. Other heavy atoms, S, Cl, and Fe atoms, are marked on the sphere. Molecular topologies with the coordinates are generated by using Avogadro software [412].

Despite the success of imatinib in chronic myeloid leukemia [137] (Fig 2A), and subsequently in gastrointestinal stromal tumors [138], which propelled optimism (OSI Pharmaceuticals 2002 Annual Report), progress is slow and stymied by failures. The concept of precision medicine is attractive; but the challenges are daunting. Among these are the multiple genes that can be involved, as well as the observations that tumors evolve to accumulate additional genomic alterations, and that targeting a specific actionable mutation may only be moderately effective. Data science aims to uncover these mutations through broad sequencing of samples, and statistical analyses correlate these with clinical observations. Within this framework, identifying predominant conformational states of the actionable driver mutations may only partially alleviate the dilemma; detecting which mutations, or combinations of mutations, that do not fall into the statistically actionable definition can also shift the landscape from the inactive to the active states (or vice versa) is a laudable aim.

## The complex frameworks of precision oncology

Cancer genomes are complex. Precision oncology tailors ways to surmount this complexity and achieve increased precision [139]. The key question it strives to surmount is how. The clinical view is that laid-out frameworks should contend with validation. It advocates increasingly complex diagnostic tests, innovative methods for evaluating efficacy, and review of what evidence should be considered adequate, especially for patients with genomic abnormalities whose clinical implications are unclear. The clinics endorse and urge large-scale profiling of advanced-stage tumors to recognize genomic alterations and large cohorts of patients to support therapeutic strategies, despite this contradicting the basic patient-tailored tenet of precision medicine. An additional element that the clinics contend should be rectified is the absence of databases classifying the risks and possible advantages of a specific plan for the patient. The clinics also recognize that a tissue-specific diagnosis requires understanding of cancer biology, clearly defined driver alterations, and drug combinations. In those examples above of successful clinical trials (for example, trastuzumab in HER2-positive breast cancer [140, 141] (Fig 1A), erlotinib and gefitinib in non-small-cell lung cancer with epidermal growth factor receptor (EGFR) mutations [142, 143] (Fig 1B), imatinib in Bcr-abl1-fusion-positive chronic myeloid leukaemia [144–146] (Fig 2A), and vemurafenib and dabrafenib in advanced-stage melanoma with B-Raf^V600E^ mutation [147] (Fig 2B and 2C)), there was no conflict between the essence of the trials and the definition of precision oncology. The genomic alterations were diagnosed in many patients with these cancers, along with the markers and companion diagnostic tests.

Because tumors are heterogeneous, include genetically divergent subclones, and with time acquire drug resistance, frameworks that target a single mutation may not work long term, which argues for combinatorial drug regimes. This however has its own concerns, because drug combinations increase toxicity, including that arising from drug—drug interactions, which may require dose modulation, potentially limiting their effectiveness [148, 149]. The possible presence of subclones, especially in advanced stages, as well as tumor evolution that may have been influenced by prior treatments, also argue for genomic sequencing of multiple biopsy samples. In line with this, only a minority of treated patients show clinical improvement, and in those cases, it is only for a short time.

A framework for precision oncology would also need to account for cancer cell metabolism. Oncogenic drivers and cancer-cell metabolism have been linked, suggesting that cancer cells may be sensitive to metabolic interventions. Oncogenes rewire cellular metabolism, and this rewiring of the metabolic interactions among tumor cells and between tumor and nontumor cells in the tumor microenvironment has been deemed druggable. For example, *KRAS* mutations drive metabolic alterations in colorectal cancer contributing to cell adaptation to glutamine depletion by up-regulation of asparagine synthetase [150]. Targeting nutrient transport and utilization can critically affect the tumor cell energetic needs [151]. However, the intricacy of the tumor microenvironment and the dynamic circadian metabolism question the precision of targeting cancer metabolism. Nonetheless, recent progress has raised hope that the tumor microenvironment may be precisely targeted [152].

The overall complexity challenges the clinics; it also challenges our community as to how to devise reasonable frameworks to make a difference. In one example of this complexity, lung adenocarcinoma histopathology was classified by metastatic potential and survival; however, in the absence of molecular profiles, the underlying explanation has been unclear. Subtypes mutational screening of the *EGFR*, *KRAS*, and *BRAF* genes revealed that *KRAS* and *BRAF* mutants are more abundant in higher grades, but *EGFR* driver mutations appear in all subtypes pointing to the difficulties in prognostically identifying key mutations [51, 153]. Genome-wide gene copy number aberrations and somatic mutation statistics suggest that cancer types emerged through distinct pathways. Therefore, identification of aberrations that impact key signaling pathways in cancer cells is a necessary component in development of frameworks for targeted therapeutics. Furthermore, the oncogene addiction concept, supported by observations on human cancer cell lines, mouse tumor models, and clinical cancer studies [154, 155], argues that signaling that emerges because of single constitutively activated protein can be adaptively rewired, which suggests that a single transformed oncogene can have disastrous consequences on the cancer cell.

**Fig 3:**
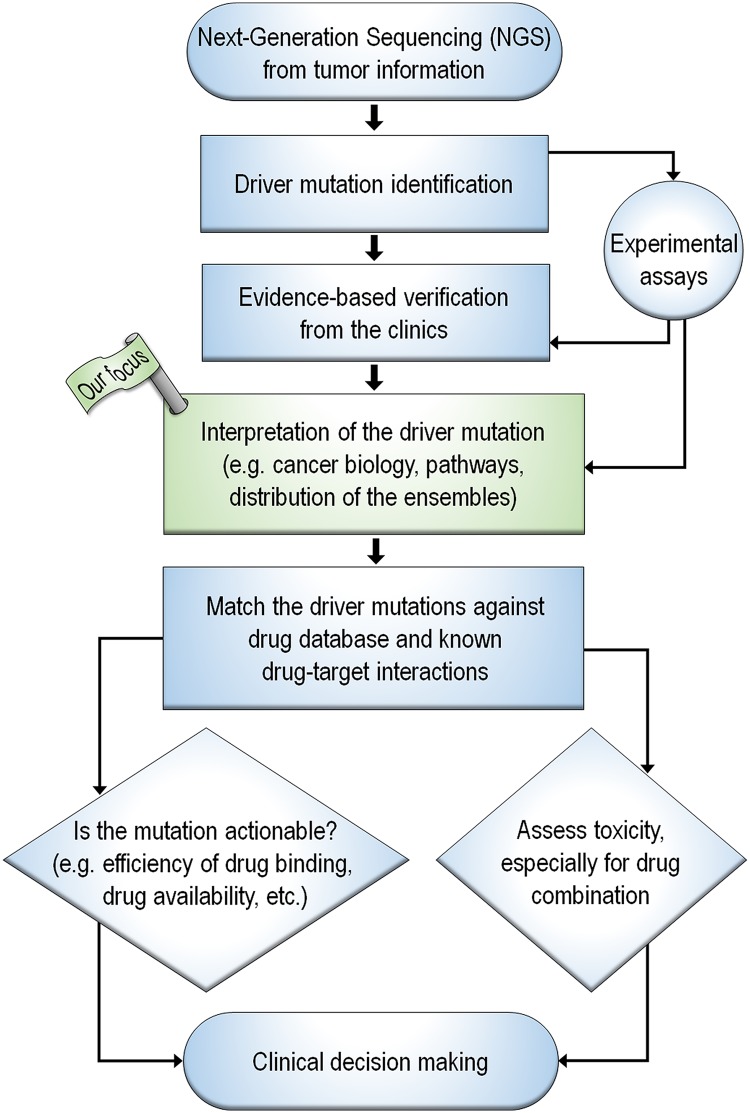
A flowchart representing the comprehensive framework to precision oncology. Here, our focus is the process of the interpretation of the driver mutation using the databases in cancer biology, cancer pathways, and the distribution of the ensembles of protein conformation. The next process to match the driver mutations against drug databases and known drug-target interactions is not discussed in this paper.

The broad range of potential driver mutations, their diverse molecular environments, and the multiple possible targets reinforce the perception that charting a path forward is fraught with doubts. The first step should enable converting batches of raw sequencing data into interpretable results; in its absence, substantial collected data, which may be related to the clinical or research question [3, 156] may not be considered. Even though clinical oncology—related bioinformatics methods and software are being developed [157], the dynamic multifactorial environment, coupled with the need for rapid, highly reproducible and robust procedures make a comprehensive bioinformatics toolkit difficult to achieve. A workflow of a molecular tumor board, which includes bioinformatics tools ranging from the analysis of profiling data to automated formulation of clinical report, has been described and implemented in several clinical trials, helping in the treatment decision-making. Even though such software support provides treatment recommendations, and bioinformatics tools and data platforms are essential to complement advances in precision oncology, their underlying foundation is restricted and fall short of what is required. We believe that one key missing component is a better understanding of the biology. Insight into the underlying biological mechanisms will help in better defining “actionability” for molecular targets [158]. The complexity of the framework is further amplified by intercellular signaling and cellular heterogeneity. A comprehensive framework of precision oncology requires an integrative, holistic approach (Fig 3). Within this fabric, multiscale approaches to systems biology may help address the competing and reciprocal nature of processes underlying cancer.

## The concept of driver mutations

Effective medical care requires accurate information. Current treatment is mostly based on identification of the patient’s driver mutations [51, 85]. Classically, a mutation is catalogued as either a driver or a passenger. Passenger mutations are taken as unrelated to the disease and thus are not considered actionable; driver mutations are. A major question has been how to distinguish between a passenger mutation and a driver mutation. This question is particularly challenging given the heterogeneity of somatic mutations in tumor cells, as well as practical considerations related to sample collection, which include populations of normal and tumor cells [69, 159]. The clonal theory of cancer [160] postulates that all cells in the tumor evolved from a single stem cell and that the driver mutation arose in that cell and propagated in successive clonal expansions. Three strategies have been proposed to define driver mutations in genomic data: (i) detection of frequent mutations through sequence analysis; (ii) predicting the functional influence of the mutation (i.e., activation and/or inactivation); and (iii) the impact of the mutation and combinations of driver mutations on signaling pathways and statistical correlations. The output may depend on how the background mutation rate (BMR) is computed and analyzed. The BMR varies not only across the genome of a given patient but also across patients and tumor types [161]. Because computing somatic mutational frequencies (normalized by that expected by chance) appears the most straightforward as compared to functional and cellular network impact assessments, most studies adopt this strategy to distinguish driver mutations from passenger mutations. In the simplest approach, the BMR of a gene is assumed as known, and the probability of passenger mutations is then estimated [162, 163]. However, BMR and mutational frequencies estimates are inaccurate and error prone. Therefore, the resulting inaccurate classification of the mutations can lead to false positives. Heuristic approaches based on protein sequences, i.e., the so-called 20/20 rule [46] classifies a gene as an oncogene if over 20% of its mutations are at a certain residue [164]. Clustering of mutations not only in protein sequences [165] but also in structures [166] have also been proposed, as well as other driver gene identification approaches [167]. Protein structural features included in the analysis can consist of predicted amino acid propensities for secondary structure, solvent accessibility, backbone flexibility, and more [45]. As examples of methods using genomic sequences and structures, cancer-specific high-throughput annotation of somatic mutations (CHASM) exploits supervised machine learning to prioritize somatic missense mutations detected in tumor sequencing [168]. CHASM trains a random forest classifier on driver mutations from the catalogue of somatic mutations in cancer (COSMIC) databases. The nucleotide substitutions that are detected in the tumor are used to identify passenger mutations. A nonsynonymous mutation is described by several quantitative features and physiochemical properties, scores obtained from multiple sequence alignments (of the protein or the DNA), residue composition in the sequence window where it falls, local structural characteristics, and more. Following the ranking of the predicted driver mutations, based on vectors of features, CHASM can predict how the mutation acts in tumorigenesis. The subsequently developed cancer-related analysis of variants toolkit (CRAVAT) is a suite of tools to interpret nonsynonymous mutations, including their mapping, annotation, impact, interpretation, and possible structural consequences [169]. Some software tools, such as Quaternary Protein Amino acid Clustering (QuartPAC) [170], identify statistically significant mutational clustering in 3D protein space, i.e., energy states, through combining available protein structures and mutational data in the COSMIC database. Recently, ParsSNP, an unsupervised functional impact predictor that is guided by parsimony was introduced [171]. ParsSNP identified many known and likely driver mutations that were missed by other methods. A detailed list of currently available tools for personalized cancer medicine is provided in Table 1.

**Table 1:** Computational and bioinformatics tools for personalized cancer medicine.

Names	Description	Website	References
**Somatic mutation callers**			
MuTect	Sensitive detection of somatic point mutations from exome and genome sequencing data.	https://github.com/broadinstitute/mutect	[413]
GATK	Detection of variants from high-throughput sequencing data.	https://software.broadinstitute.org/gatk/	[414]
VarScan	Somatic mutation and copy number alteration detection from exome sequencing data.	http://varscan.sourceforge.net/	[415]
Strelka	Somatic small-variant detection from sequenced tumor-normal sample pairs.	https://github.com/Illumina/strelka	[416]
**Sequence-based predicting functional consequences of genomic variants**			
PolyPhen-2	A popular tool for predicting the functional impacts of protein sequence variants based on naive Bayes classifiers.	http://genetics.bwh.harvard.edu/pph2/	[417]
SIFT	A popular tool for predicting the biological effect of missense variations using protein sequence homology.	http://sift.bii.a-star.edu.sg/	[418]
CHASM & SNVbox	Python and Cþþ programs for predicting cancer-related mutations based on their tumorigenic impact.	http://wiki.chasmsoftware.org/	[419]
MutationAssessor	Predicting functional impact scores based on evolutionary conservation patterns.	http://mutationassessor.org/r3/	[93]
CADD	A tool for scoring the deleteriousness of single nucleotide variants as well as insertion and/or deletions variants.	http://cadd.gs.washington.edu/	[217, 420]
**Mutational Clustering tools on Protein Structures**			
CLUMPS	Assess the significance of mutational clustering in a given 3D structure.	NA	[421]
HotSpot3D	Detect mutation—mutation and mutation—drug clusters using 3D protein structures.	https://github.com/ding-lab/hotspot3d	[422]
SGDriver	Detect mutation clustering on protein—ligand binding site residues using a Bayes inference statistical framework.	NA	[423]
AlloDriver	Detect mutation clustering that alters dysregulation of protein allosteric sites.	NA	[186]
KNMPx	Detect mutation clustering that rewires phosphorylation-related signaling networks and drug sensitivity and/or resistance.	NA	[193]
ReKINect	Detect network-attacking mutations in phosphorylation-based signaling networks.	http://rekinect.science/	[226]
Cancer3D	Identify potential cancer drivers or pharmacogenomic biomarkers using protein structure information.	http://cancer3d.org/	[424]
**Sequence-based predicting cancer driver genes**			
MutSig & MutSigCV	Prediction of significantly mutated genes from heterogenous tumor exome sequencing data.	http://software.broadinstitute.org/cancer/software/genepattern/modules/docs/MutSigCV	[425]
MSEA	Prediction of hotspots using mutation set enrichment analysis.	NA	[426]
20/20 rule	Evaluating the proportion of inactivating mutation and recurrent missense mutations in a gene.	NA	[46]
20/20+	A machine-learning based, 20/20 rule by integrating multiple features of positive selection.	NA	[84]
ActiveDriver	Finding cancer driver proteins with enriched mutations in post-translational modification sites.	https://cran.r-project.org/web/packages/ActiveDriver/	[427]
OncodriverFM & OncodriverFML	Identifying driver genes based on functional impact bias.	http://bg.upf.edu/oncodrive	[428]
OncodriverClust	Identify driver genes with a significant bias towards mutation clustering within the protein sequence.	http://bg.upf.edu/oncodriveclust	[429]
MuSiC	A driver gene detection framework by quantifying correlation of clinical data with mutation sites, affected genes and pathways.	http://gmt.genome.wustl.edu/	[430]
**Network-/pathway-based prediction of cancer driver genes**			
HotNet2	A tool for detecting mutated subnetworks in cancer using an insulated heat diffusing algorithm.	http://compbio.cs.brown.edu/projects/hotnet2/	[431]
NBS	A somatic mutation network-based approach for stratifying tumor mutations.	http://chianti.ucsd.edu/~mhofree/NBS/	[432]
DawnRank	Prediction of cancer genes in a single patient based on the PageRank algorithm.	http://bioen-compbio.bioen.illinois.edu/DawnRank/	[433]
VarWalker	Identifying putative cancer genes using personalized mutation network analysis.	https://bioinfo.uth.edu/VarWalker.html	[434]
DriverNet	Predicting driver mutations by estimating their effect on mRNA expression networks.	http://compbio.bccrc.ca/software/drivernet/	[435]
TieDIE	Predicting cancer mutated subnetworks using a network-based diffusion algorithm.	https://sysbiowiki.soe.ucsc.edu/tiedie	[436]

Analysis of clusters, or spatial proximity of rare mutations, in 10,000 tumor exomes detected over 3,000 rare mutations and catalogued them as potentially driver mutations [172]. Among them, those in *RAC1* and *MAP2K1* were validated. These not only confirm the paradigm that statistics can identify driver mutations but also that driver mutations often cluster. This tendency for proximal associations can suggest cooperativity, enhancing their effects, similar to the observation of a tendency of residue hot spots to cluster into “hot regions” [173, 174]. This comprehensive study analyzed over a million somatic missense mutations in 11,119 human tumors in 32,445 protein structures from 7,390 genes. Well-established drivers such as KRas^G12D^ were observed, but most residues in the clusters are rare mutations, such as KRas^D33E^ (defined as observed in <0.1% of the sample) [175]. Many are oncoprotein such as KRas (*KRAS*), B-Raf (*BRAF*), and p53 (*TP53*). All are cataloged and available. The authors note that in their analysis that most of these rare mutations appear to be passenger mutations, but some may be hitherto unknown drivers [175]. Cluster analysis in protein structures also revealed rare missense driver mutations in tumor suppressors including *PTEN*, *CDH1*, and *KEAP1*, which may inactivate the proteins. *PTEN* tumor suppressor yielded 15 clusters totaling 48 residues (with 2 established drivers and 46 rare mutations). All are around the phosphatase catalytic core motif [176]. Because the respective experimental structures are available, these can serve as a benchmark for testing methods for predictions of rare driver mutations, or latent driver mutations, exploiting conformational sampling and analysis, including allostery.

When mutated, hundreds of genes can drive cancer development. However, a surprisingly small number of driver mutations can already suffice. For lung and colorectal cancers, mathematical modeling has estimated the number to be three [177]. However, other estimates suggest 1 to 10 mutations with the number of mutations driving cancer varying considerably across different cancer types; four mutations per patient on average drive liver cancers, whereas colorectal cancers typically require about 10 [178]. Furthermore, the genes vary in the proportion of drivers versus passengers.

## Interpreting variants in precision genomic oncology

To date, interpretation of the mutational landscape is essentially based on software packages. Among these is mapping genetic variations to 3D protein structures [179]. Ultimately, accurate translation of personal genomics to precision medicine requires factual interpretation of numerous genetic individual variants. Even though the structures of proteins with nonsynonymous mutations can be predicted, the functional implications may or may not be clear. The consequences of mutations in enzyme active sites or at protein—protein interfaces are more straightforward to interpret. In one example, mapping glioblastoma missense mutations on the human structural protein interactome observed that even though some missense mutations overstabilize protein complexes, most of them are destabilizing. In particular, mutations on interfaces indicated larger changes of amino acid physico-chemical properties than those occurring elsewhere. This allowed identification of potential driver genes and additional possible cancer [180]. This, however, is often not the case, and allosteric effects are often at play [99, 181–189]. Millions of genetic variants have been catalogued in genomic databases, and the Protein Data Bank (PDB) documents the structures of thousands. If a homologous structure is available, it is now straight forward to model the others. This strategy permits mapping the mutations, testing how these mutations affect protein stability or its interactions, and thereby guide drug discovery.

Recently, a comprehensive and critical overview and resource compilation for interpreting variants in precision genomic oncology applications has been published [24]. Small variants are defined as single nucleotide variants or short (up to approximately 20 base pairs) insertions and/or deletions. The authors outline a precision genomic oncology application scheme to high-throughput sequencing [190]. Their aim is to facilitate bedside decisions, while at the same time emphasizing the challenge embedded in the interpretation of individual variants. The essential question they aim to address is how to integrate statistical identification of variants with biological data. Toward this, they compile and develop variant catalogs, databases of actionable mutational variants, and software and tools for variant annotation. They also assemble databases for predicting the impact on protein function. They detail how to exploit this collection, either in individual cases or integrated, to be used for interpreting clinical results and acting on them at the bedside. Drug—target interaction resources have also been compiled into a so-called Cancer Targetome along with assessment of supporting evidence [191].

Molecular alterations that act as drivers of cancer can also be ranked by integrating genomic and transcriptomic profiles. One example is HIT’nDRIVE. Its goal is to identify a set of patient-specific, sequence-altered genes that, when combined, can alter dysregulated transcripts [192]. When applied to 2,200 tumors from four types of cancer, it discovered potentially actionable driver genes and predicted drug efficacy. In a different approach, KNMPx identifies oncogenic alterations that rewire phosphorylation-related signaling networks [193]. This method considers the functional consequences of somatic missense mutations at phosphorylation sites observed in pancancer analysis. By integrating 746,631 missense mutations in approximately 5,000 tumor samples from 16 cancer types, KNMPx obtained 170,000 nonredundant phosphorylation sites in 18,610 proteins with 47 enriched with missense mutations at their phosphorylation sites. The mutations identified by KNMPx in tissue-specific kinase-substrate interaction modules were observed to relate to survival and sensitivity and/or resistance of inhibitors targeting epidermal growth factor (EGF), mitogen-activated protein kinase (MAPK), phosphatidylinositol-4,5-bisphosphate 3-kinase (PI3K), mammalian target of rapamycin (mTOR), and Wnt signaling pathways. Integrating genomic and transcriptomic analyses benefits precision oncology [5], as does the integration of cancer genomics and clinical oncology [194]. To date, there are several computational approaches based on such integration to detect drivers and potential genetically suspect regions (e.g., [14, 195, 196]). Driver mutations affecting CBL ubiquitin ligase activation were identified through a new approach [197] and a method, MutaBind, for estimating and interpreting the effects of sequence variants on protein—protein interactions was developed as well as cellular networks [198].

Genomic analysis can identify patients with high tumor mutational burden as well as recurrent regulatory mutations, such as in the case of mismatch repair endonuclease *PMS2* [199]. Personal genomics can also help in classification and thereby precision treatment. One recent example is pediatric oncology [200]. Pediatric high-grade gliomas (HGGs) have been treated following adult regimens with minimal clinical benefit [201, 202]. Even though phenotypically indistinguishable, next-generation sequencing unraveled alterations different from those of adults [203], including somatic mutations in histone genes *H3F3A* (replication-independent histone 3 variant H3.3) and histone 3 [204].

Altogether, interpreting gene copy number, transcriptome and proteome levels, epigenetic alterations, somatic mutational statistics, all within the framework of cancer types, as well the effects of drugs, are key hurdles challenging the investigator.

## Software tools are important but insufficient

The need to predict and annotate mutational variants in individual genomes to accurately identify molecular targets is pressing. The surging tide of big data, machine learning, and data analytics has spawned the sweeping impression that efficient and capable programs can resolve most questions, and it may not be necessary to go deeper into the underlying biology [205]. Recent surveys vindicate the increasing appreciation and passion for powerful software development. Among the computational needs are high-performance computing, bioinformatics, workflows, and up-to-date analysis codes and/or servers and data integration with the belief that data science components and skills are necessary for in-depth understanding of processes within and between cells [206]. Machine learning can be a shortcut to discover correlations between processes at different scales. However, in biology and medicine approaches based solely on big data forsaking a conceptual account can fail [205]. Regardless of their sophistication, know-how, and cleverness, eventually, artificial intelligence methods work by fitting. If the data is “big,” they can be statistically reliable. However, when trying to capture and predict rare events, they may fail. Biological knowledge and conceptual insight should integrate with big data—based methods to obtain more accurate and dependable conclusions on complex systems, like those needed here.

Software can analyze genomic, proteomic, microarray, and clinical data [206–210]. If the data is massive, it is possible to correlate the results with experimental observations [211, 212]; however, results based wholly on statistics cannot explain why the correlations are observed. A statistical result is the outcome of the many parameters that go into the training of the software. Even though strategies like bootstrapping and leave-one-out cross-validation can help in pinpointing the key variables, the multiple parameters still cloud the interpretation, diminishing the accuracy of predictive precision medicine [213]. However, powerful genomics, transcriptomics, and proteomics software can couple with computational, theoretical, and experimental structural biology. This compelling combination can more effectively unravel the underlying mechanisms of alterations in protein coding regions, and their interactions with other proteins, lipids, DNA and RNA, and drugs. If resources are available, computations can reach mechanistic details that are currently challenging for experiments to deliver. Integrative multiscale approaches can increasingly be applied across a range of scales to offer and test theories and concepts, and notably, the predictions and observations that they make are quantified.

Precision oncology would benefit from merging genetic data and analysis with the underlying structural foundation. Structures not only help understand genetic aberrations, but they can also innovate hypotheses and offer mechanisms, helping in more accurate tailored treatments. Methods that predict the effects of mutations on protein stability, the hydrogen bond network, pH dependence, conformational dynamics, and protein function have been usefully compiled [214]. Below, we discuss their potential effects on the free energy landscape of the proteins, how driver mutations can affect them as compared to passenger mutations, and introduce the concept of “latent drivers.” Annotation and prediction methods based on structural and biophysical information have also been compiled [215].

## Functional assays of actionable variants: Tip of the iceberg

Bioinformatics analysis and computational strategies offer important information for identifying hot spot mutations and significantly mutated genes in cancer. However, these analyses cannot provide definitive proof of causal mechanisms to permit transfer of basic discoveries directly to the point of care. An experimental measure of functional consequences of mutations or cancer genes is crucial to decipher the tumorigenic mechanisms and to identify novel therapeutic targets. Here, we briefly describe several experimental strategies to explore the functional consequences of mutations altering tumorigenesis, disease progression, and drug responses.

## Protein expression altered by somatic mutations

**Fig 4:**
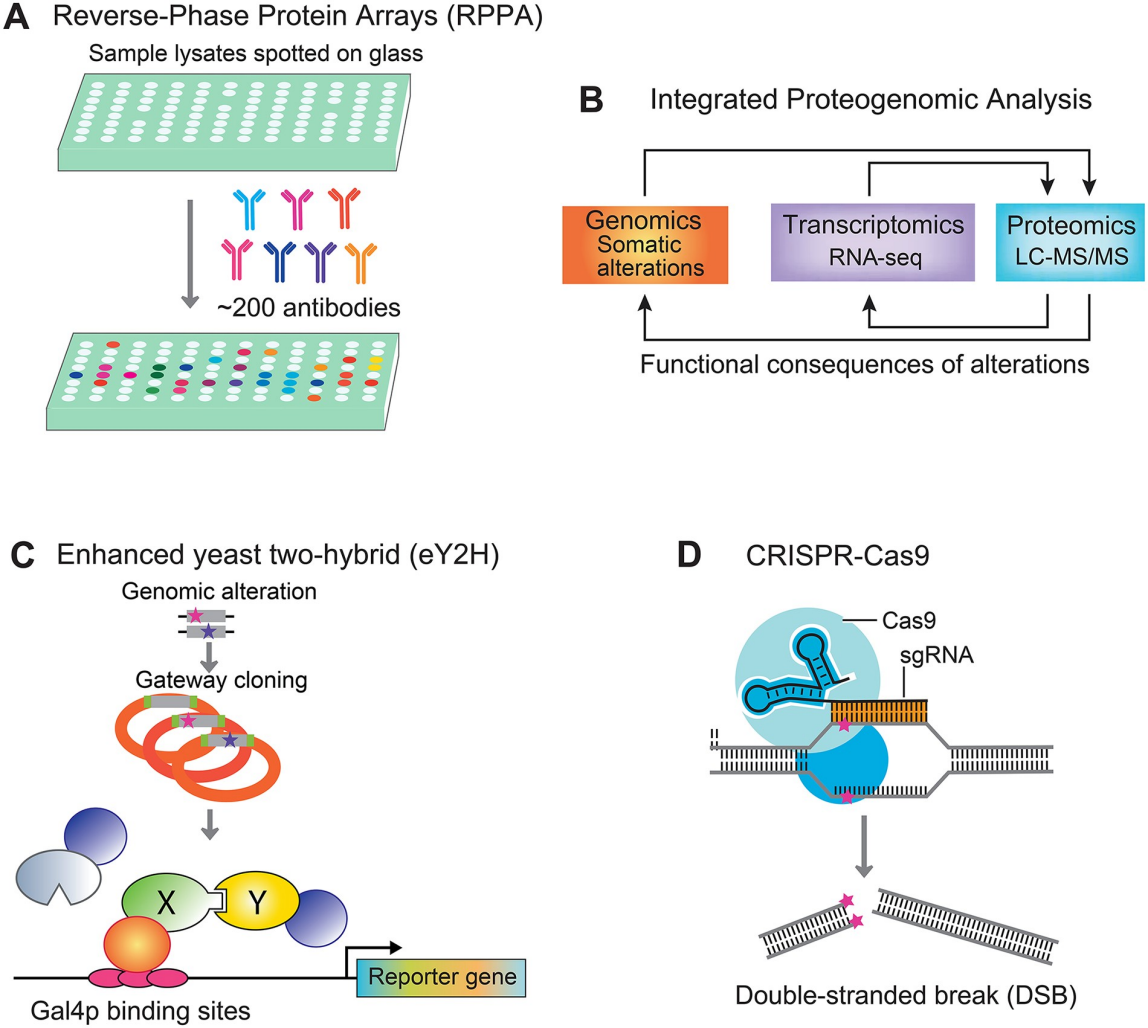
Examples of experimental strategies to explore the functional consequences of mutations altering tumorigenesis, disease progression, and drug responses. These include (A) RPPA technology, (B) integrated proteogenomic analysis, (C) high-throughput gateway-compatible enhanced yeast two-hybrid, and (D) CRISPR-Cas9 genome editing. LC-MS/MS, liquid chromatography—tandem mass spectrometry; RPPA, reverse-phase protein assay.

Several antibody-based pipelines have been used to measure protein expression profiles altered by somatic mutations in cancer. Reverse-phase protein assay (RPPA) technology is a common protein microarray that uses antibodies to measure the relative expression levels of proteins in tissues or cells (Fig 4A). Ng and colleagues developed a moderate-throughput functional pipeline to inspect the functional impact of over 1,000 genomic alterations, including gene amplifications, point mutations, indels, and gene fusions [216]. They used two growth factor—dependent cell models and functional proteomic signaling profiles (RPPA) for selected alterations. They showed that this functional assay approach can be used to identify weak drivers. However, most RPPA datasets commonly involve approximately 200 antibodies that measure the total proteins or specific protein post-translational modifications, such as phosphorylation. Alvarez and colleagues reported a network-based inference algorithm, a virtual inference of protein activity by enriched regulon analysis (VIPER), for functional characterization of somatic mutations in cancer [217]. They applied VIPER to investigate the functional relevance of genetic alterations in regulatory proteins in The Cancer Genome Atlas (TCGA) samples. Combining in vitro assays, they demonstrated that VIPER outperformed traditional mutational analysis in evaluating the sensitivity of targeted cancer agents by inferring protein activity. Inferring protein expression (activity) following genomic alterations is increasingly emerging as an optimal biomarker for identifying therapeutic targets in cancer. However, mRNA expression cannot reliably predict protein abundance differences among tumors [218]. Proteogenomics, which integrates proteomics, genomics, and transcriptomics, offer novel tools for understanding cancer biology [219]. Zhang and colleagues reported the first proteogenomic analysis of 95 TCGA colon tumor samples using liquid chromatography—tandem mass spectrometry (LC-MS/MS)-based shotgun proteomic approach [218]. Via integration of sequencing, transcriptomic, and methylation data available from TCGA, they showed that in colon cancer, proteogenomic analysis enabled finding novel candidate driver genes compared to a sequencing-only approach [218]. Subsequent proteogenomic analysis helps elucidate the functional consequences of somatic alterations and predicts clinical outcomes in multiple cancer types, including breast cancer [220] and high-grade serous ovarian cancer [221]. In summary, integrated proteogenomic analysis (Fig 4B) offers novel insights into genomic alterations, including novel biomarkers and therapeutic targets for understanding cancer biology and treating cancer, such as that shown by the Clinical Proteomic Tumor Analysis Consortium (CPTAC) project [218, 220, 221].

## Cellular networks can be modified by somatic alterations

Genomic alterations can change cellular network perturbations [222, 223], such as protein—protein interactions (PPIs), protein—DNA interactions, and protein—metabolite interactions [224]. Several large-scale functional assay platforms have been used to elucidate these interaction changes. For example, Sahni and colleagues showed that most disease mutations do not impair protein folding or stability using a luminescence-based mammalian interactome mapping (LUMIER) assay [225]. However, using the high-throughput gateway-compatible enhanced yeast two-hybrid (HT-eY2H) (Fig 4C), orthogonal in vivo Gaussia princeps luciferase protein complementation (GPC), and the enhanced yeast one-hybrid (eY1H) assays, they found surprisingly widespread disease-specific perturbations of PPIs or protein—DNA interactions [225]. For example, using the integrated assays, they observed that the EGFR L858R mutant can interact with heat shock protein 90 (HSP90) [225]. In addition to PPIs, protein post-translational-modification signaling network (i.e., phosphorylation sites) are often altered in cancer. Creixell and colleagues reported a novel algorithm, ReKINect, to detect network-attacking mutations in phosphorylation-based signaling networks [226]. Using ReKINect, their quantitative analysis of (phospho-)proteomes of five ovarian cancer cell lines and other available tumor sequencing profiles experimentally identified several network-attacking mutations that alter specific switches, analogous to de novo appearance of kinases within the kinome [226]. In summary, the experimentally identified network perturbations altered by genomic changes offer novel diagnostic tools and therapeutic targets for more personalized treatments (i.e., PPI inhibitors).

## CRISPR-Cas9 genome editing

Owing to their high efficacy (i.e., low off-target effects) and ease of use compared with other tools, clustered regularly interspaced short palindromic repeat-associated 9 (CRISPR-Cas9) systems have become the most popular genome-editing tool (Fig 4D). CRISPR-Cas9 offers multiple ways to functionally inspect whether a mutation is a causal tumorigenic mechanism. In principle, CRISPR-Cas9 knock-in of alternate mutations (i.e., alleles of missense mutations) into cellular or animal models, generates wild-type and variant models that are isogenic, i.e., of matched genetic background. As the only difference between the models is with respect to the missense mutations, in principle any tumorigenic differences observed between the models can be attributed to the single missense mutations. For example, homology-directed repair-mediated *KRAS* G12D mutations using CRISPR-Cas9 lead to macroscopic tumors with adenocarcinoma pathology, revealing that Cas9 mice empower a wide range of biological and disease modeling applications [227]. Recently, a guide RNA and catalytically impaired CRISPR-Cas9 were used to convert A-T base pairs to G-C in the genome, enabling the editing of single point mutations without inducing double-stranded DNA breaks [228]. Gebler and colleagues first reported the inactivation of cancer mutations using CRISPR-Cas9 that combines comprehensive single-guide RNA (sgRNA) design and an efficient reporter assay [229]. However, it is challenging to design unique sgRNAs for every mutation using additional orthogonal CRISPR-Cas systems, and control the subsequent repair after Cas9-mediated DNA cleavage. Moreover, a potential risk of oncogenic phenotypes owing to possible off-target cleavage has to be evaluated in vivo prior to a clinical setting in cancer patients. The resulting large-scale functional data can be combined with computational strategies and clinical knowledge for the development of integrative approaches for accurate pathogenicity predictions.

## An ensemble view of precision oncology

### An introduction to the protein landscape

**Fig 5:**
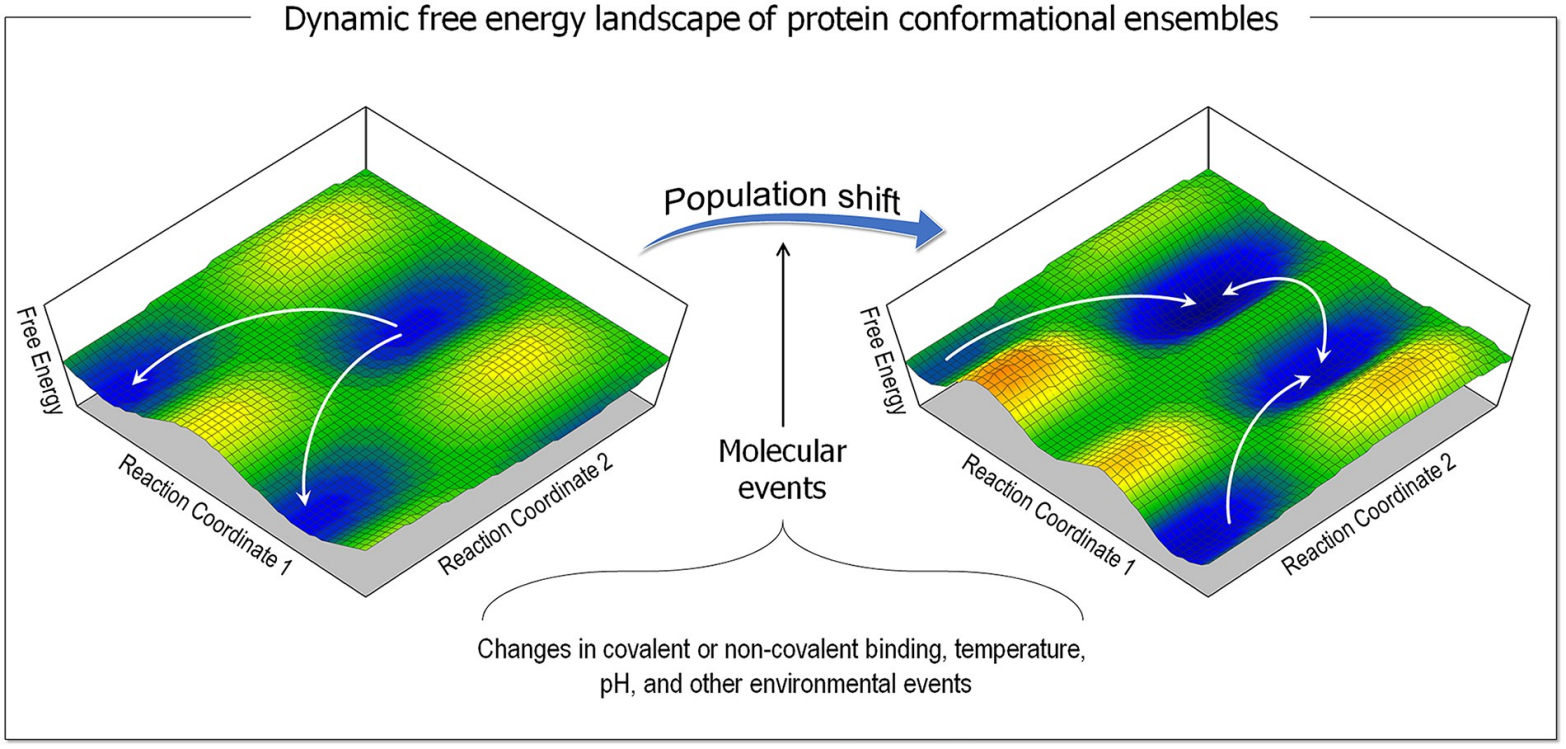
An example shown for the dynamic landscape of the free energy surface representing the protein conformational ensembles. Molecular events result in the population shift of the conformational ensembles, redistributing the populations of the states.

A protein executes its function when it populates a distinct conformational state. The landmark work of Frauenfelder, Sligar, and Wolynes [230] portrayed protein ensembles in terms of the free energy landscape. The landscape idea is compelling because it describes the mapping of all possible conformations, native and nonnative, that the protein can populate as a function of their corresponding energy levels, on a two (or three)-dimensional Cartesian coordinate systems. The picture that the landscape paints of the protein states is powerful because it clarifies the physical basis of the ensemble around the native state of the protein. That picture captures the ensemble under a certain set of conditions. However, the picture is static; as such it is unable to account for biological function, which is based on cooperativity. This led us to propose that the landscape is dynamic, and that the relative distributions of the conformational states can change in response to intra- and extramolecular events, and that this is the origin of cooperativity, i.e., allostery [231, 232]. These events can be binding (noncovalent, e.g., ligands, lipids, proteins, water, and nucleic acids or covalent, e.g., mutations, post-translational modifications [PTMs], such as phosphorylation, farnesylation, palmitoylation, and ubiquitination) [233], changes in temperature, pH, and other environmental events [234]. The outcome of these events is a shift of the populations of the states, which we termed “population shift” (Fig 5). This results in redistributions of the populations of the states. Therefore, an allosteric effector exerts its functional consequence by shifting the ensemble from a highly populated inactive state to a highly populated active state or vice versa [235].

Allostery is vital for biological processes at all stages. We proposed a unified view of allostery, which integrates three perspectives: thermodynamics, free energy landscape of population shift, and structural—all with exactly the same allosteric descriptors [236]. The novelty of the unified perspective rests in its linkage of these elements. The thermodynamic perspective quantified the binding of the ligand to the active (or inactive) states by assigning experimentally measurable quantities. The free energy landscape presents the population shift (Fig 5) in terms of energy indicating the relative stabilization (or destabilization) of the two biologically relevant active (or inactive) states. The third structural component, links the first two elements, the thermodynamic and the free energy landscape perspectives, pointing to the propagation pathway between the protein active and allosteric sites. In the absence of events eliciting the population shift, the pre-existing propagation pathway does not implicate allostery; it simply points to a route between the two sites that implies that they are coupled [237]. This point is important because numerous studies focus on discovering the pathways between the two sites. Further, relevant to our point here, conformational studies of allosteric driver mutations often seek to reveal pathways linking the mutation with the active site [238–241]. Also, relevant to mutational landscapes populated by allosteric driver mutations is that the dynamic free energy landscape posits that all possible conformations that a protein can populate pre-exist, including those of conformers harboring driver mutations [234, 242–244]. An allosteric effect would merely cause changes in the extent that these conformations are populated. An inactive protein populates its inactive state. When activated via an allosteric driver mutation, it conformation populates the now more stable active state.

Over the years, three hypotheses were proposed to describe the mechanism of protein binding. In the “lock and key” theory, the protein was rigid. Therefore, its binding required that its partner has a shape that matches precisely the protein’s binding site. The “induced fit” theory hypothesized that the shape of the protein when it binds to its partner can differ from the one observed in the bound state in the complex. That is, the binding process itself induces a conformational change for optimal fit. Neither of these can explain how allosteric driver mutations can exert their oncogenic effects. Both theories overlooked the physical foundation of the free energy landscape, i.e., that in solution the protein exists in a large ensemble of dynamically interconverting states, and that populations can be redistributed, as is the case in the presence of allosteric mutations (or PTMs). Based on this notion, we theorized that the binding process can be described by a “conformational selection and population shift” [231, 245, 246]. Our concept implied that rather than evolving to support new functions, as in the case of allosteric driver mutations, distinct functions can make use of pre-existing conformations, with the requirement that their population needs to be increased [247]. This change may involve breaking and/or creating new interactions, which driver mutations in cancer encode. The “conformational selection and population shift” idea explained that because the landscape is populated by many conformations, binding will take place via those which are most compatible, with a minor conformational rearrangement for optimal fit [248]. Because the binding conformers are removed from solution, the ensemble will shift (“a population shift”) toward this state to retain the equilibrium. Population shift, which redistributes the conformational states, underlies allostery and regulation. It can clarify how allosteric driver mutations can alter signaling in cancer [101, 249] and links fundamental physicochemical principles and biological processes like signaling. By constitutively increasing the population of the active conformation, allosteric driver mutations can promote oncogenic signaling.

In line with this view, strong correlations between folding and/or binding free energy changes and probability given mutations to be disease-causing were observed [250], and the effects of mutations on protein structure, dynamics, and energy landscape were reviewed in this light [251].

## The free energy landscape and driver mutations: The kinase example

**Fig 6:**
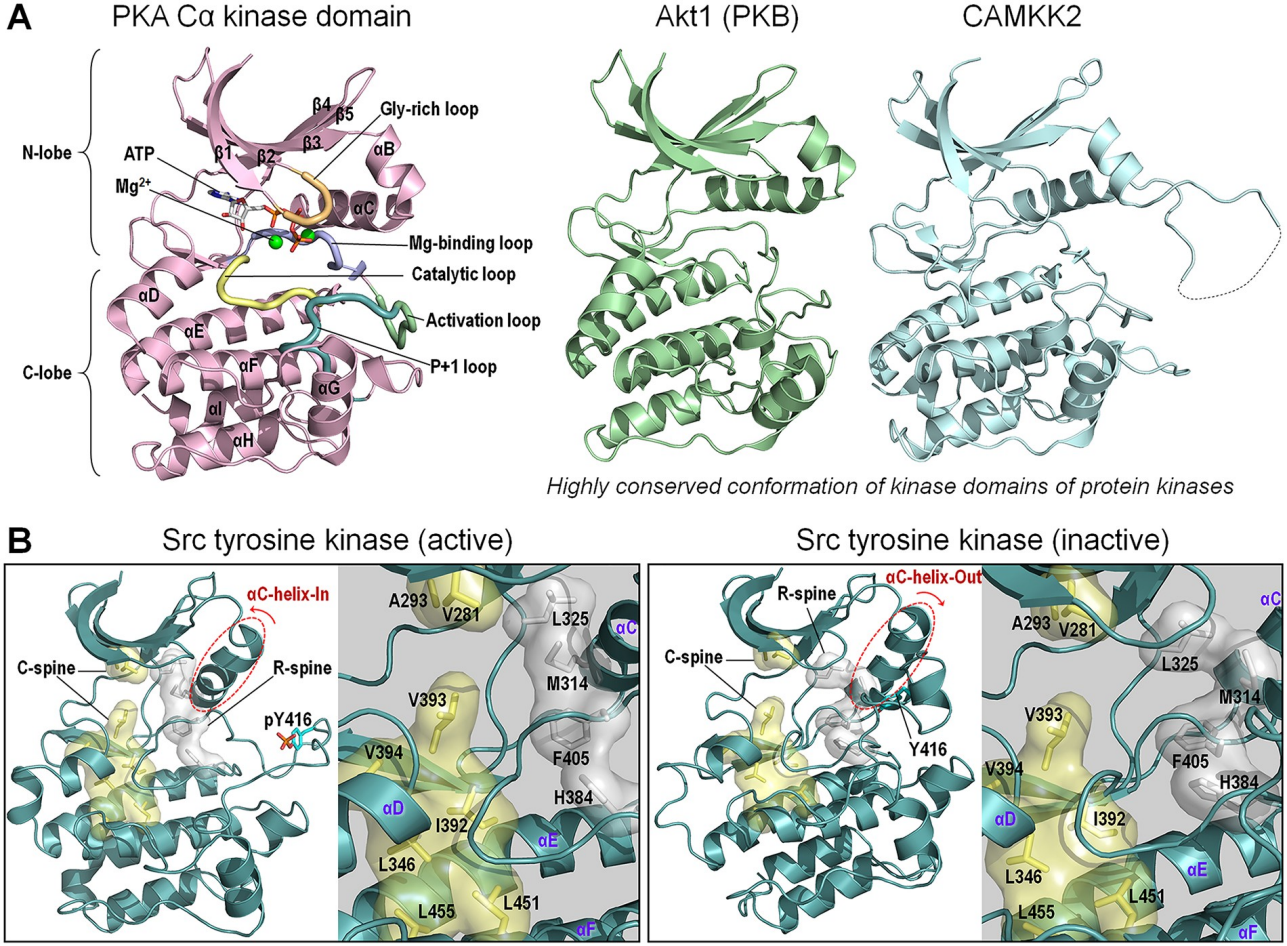
Kinase domain structures of protein kinases. (A) Crystal structure of kinase domain of cAMP-dependent PKA Cα in the active state (PDB code: 4DH3). PKA is a member of the AGC kinase family. The kinase domain conformation is highly conserved among other protein kinases. Examples are shown for crystal structures of Akt1 (PDB code: 6CCY) and CAMKK2 (PDB code: 5UYJ). Akt is known as PKB and belongs to the serine/threonine-specific protein kinase family. CAMKK2 is a member of CaM kinase family. (B) Crystal structures of kinase domain of proto-oncogene tyrosine-protein kinase Src in the active (PDB code: 1YI6) and inactive (PDB code: 2SRC) states. Src is a member of the nonreceptor tyrosine kinase family. While C-spine is preserved, R-spine is significantly distorted in the inactive Src kinase domain due to outer movement of the αC-helix. AGC, kinase group AGC; C-spine, catalytic spine; CaM, Calmodulin; CAMKK2, Calcium/Calmodulin dependent protein kinase kinase 2; cAMP, cyclic AMP; PDB, protein data bank; PKA Cα, protein kinase catalytic subunit α; PKB, protein kinase B; R-spine, regulatory spine.

Mutant kinase provides one example of the shift of the ensemble from the inactive to the constitutively active state, linking precision oncology to the free energy landscape via an allosteric driver mutation [249]. Kinases are vital to the life of the eukaryotic cell, accomplishing a broad range of functions [252]. They are also often involved in oncogenic signaling and are predominant molecular targets in precision oncology. Their catalytic action involves three steps, binding of ATP and the substrate, which has a Ser, Thr, or Tyr residue, transfer of the γ-phosphate of ATP to an accurately aligned hydroxyl group of the respective catalytic residue, and release of the phosphorylated substrate and ADP. ADP release is the rate determining step [253]. The conformational requirement of optimal phosphate transfer dictates the highly similar active conformations of the catalytic domain of all kinases [254, 255] (Fig 6A). By contrast, because different ligands bind and activate the diverse kinases, the inactive conformations vary [256].

In eukaryotic protein kinases (EPKs), hydrophobic contacts and electrostatic interactions accurately orient and coordinate the catalytic residues in the active conformation. The αF-helix organizes the conserved αC-helix, Gly-rich loop, catalytic loop and activation segment (Mg-binding loop, activation loop, and P+1 loop) through the regulatory-spine (R-spine), catalytic-spine (C-spine) [257], and electrostatic interactions (Fig 6A). Among the key features that differentiate between the two, active and inactive states, are the extended conformation of the activation loop, a specific salt-bridge and an organized R-spine observed in the active αC-helix-In but not in the inactive αC-helix-Out conformation (Fig 6B). Kinase activation commonly involves a rotation and shift of the αC-helix between these states, a conformational change that is governed by an allosteric switch. Driver mutations can control this switch. Allosteric inhibitors abutting the ATP binding site act by capturing the inactive αC-helix-Out state [258]. In many receptor tyrosine kinases (RTKs), juxtamembrane or the C-terminal can *cis*-autoinhibit catalysis. Finally, not only the structural features differentiate between the two states; the flexibility between the two lobes that are connected by a hinge linker is restrained in the active state [259–262]. Under normal physiological conditions, the catalytic core domain populates the inactive state [263]. Oncogenic driver mutations act by either stabilizing the active or disrupting interactions that stabilize the inactive conformation. Both scenarios shift the ensemble to the active state. Forty-one percent of the EGFR mutations in lung cancer present the oncogenic Leu858 driver mutation [101, 264]. This driver mutation stabilizes the αC-helix-In active conformation. By contrast, the T790M mutation in EGFR, T315I in Bcr-abl, T334I in c-Abl, T341I in Src, T670I in c-Kit (also known as CD117), and T674I in platelet-derived growth factor receptor α (PDGFRα) stabilize the hydrophobic R-spine, which destabilizes the inactive conformation (Fig 6B). The outcome is similar: the mutations allosterically switch the preferred states toward a constitutively activated kinase.

**Fig 7:**
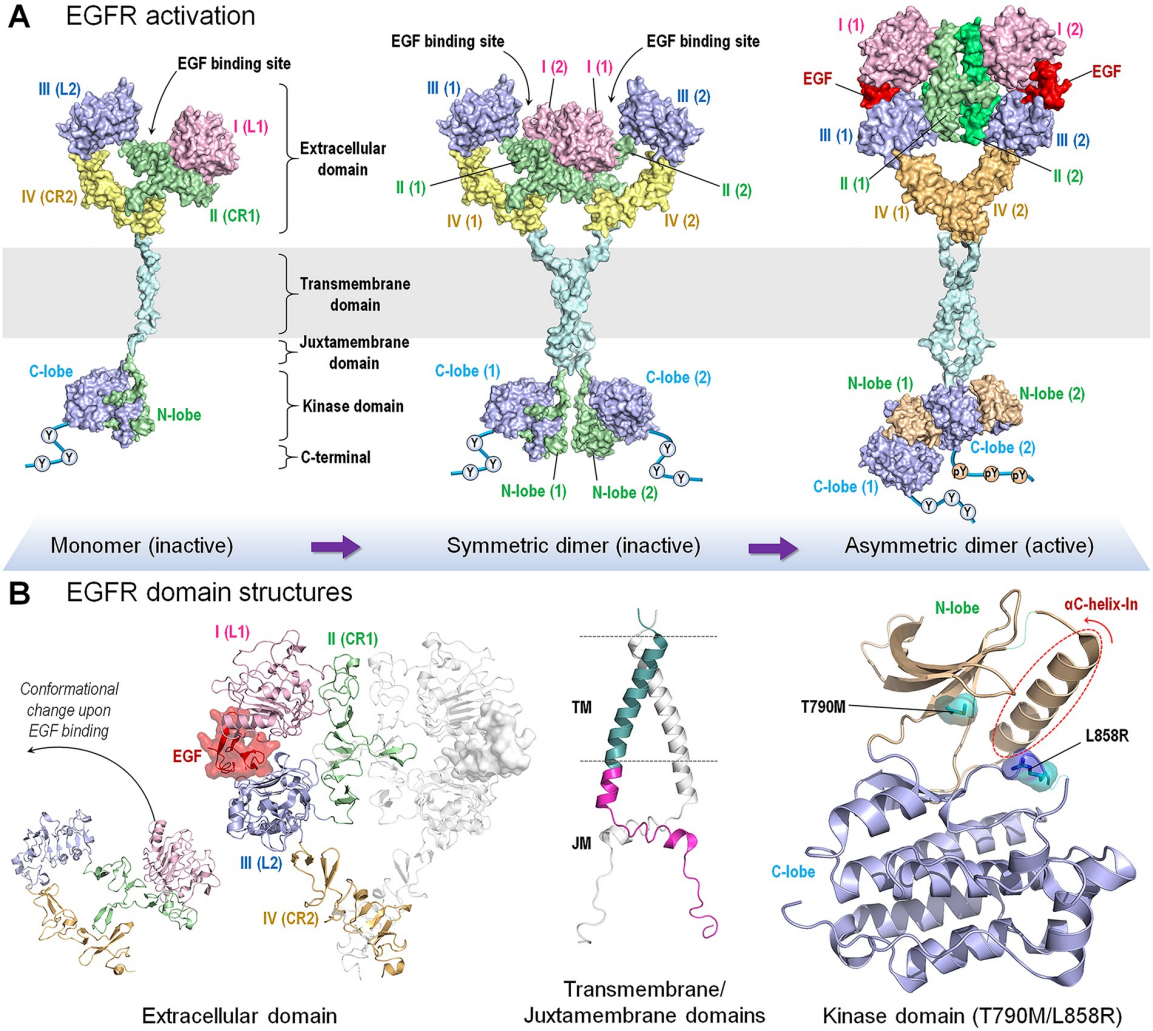
EGFR dimerization and activation. (A) Model for ligand-induced homodimerization of EGFR. The inactive EGFR monomer forms a symmetric dimer in the inactive state. Ligand binding to the extracellular domain shifts the population to the active EGFR dimer. (B) EGF or ligand binding causes an extended conformation of the extracellular domain, promoting a conformational rearrangement through the transmembrane to juxtamembrane domains [263, 270]. This results in an asymmetric assembly of the kinase domains. In the cartoons, crystal structures of extracellular domain (PDB code: 5XWD), transmembrane domain (PDB code: 5LV6), and kinase domain (PDB code: 2GS7) were used to model the inactive monomer and symmetric dimer. The active asymmetric dimer model employs crystal structures of extracellular domain (PDB code: 3NJP), transmembrane/juxtamembrane domain (PDB code: 2M20), and kinase domain (PDB code: 2GS6). Spheres in the C-terminal tail represent tyrosine (Y) and phosphorylated tyrosine (pY) residues. The mutant kinase domain cartoon employs crystal structure of kinase domain with T790M/L858R mutant (PDB code: 5EDP). EGFR, epidermal growth factor receptor; PDB, protein data bank.

EGFR can exist as a monomer, and symmetric (inactive) and asymmetric (active) dimers (Fig 7A). The autoinhibited monomer and symmetric dimer are inactive states. Under physiological conditions, activation and signaling initiate upon ligand binding to the extracellular domain (Fig 7B). This promotes a conformational rearrangement, which is transmitted through the transmembrane domain to the juxtamembrane. The resulting conformational change stabilizes the active conformation, shifting the population from the otherwise predominant inactive state toward the active asymmetric dimer. Driver mutations can mimic the physiological ligand, with a similar, albeit constitutive, signaling outcome. Driver mutations can achieve such activation through three scenarios. In the first, the T790M driver mutation, which introduces a hydrophobic residue, stabilizes the active monomer conformation through the R-spine, which increases the population of the asymmetric dimer. The L858R driver mutation works via a second scenario. This mutation replaces a hydrophobic residue by a charge in the hydrophobic core, thus abolishing interactions that stabilize the inactive state, also leading to a shift toward the active conformation. The L858R mutation therefore works by destabilizing the inactive symmetric dimer interface versus the asymmetric dimer. However, in a third scenario, NMR and simulations indicate that the L858R mutation can also adopt a second mechanism, stabilizing the active state [264]; i.e., it can stabilize the αC-helix-In conformation similar to the T790M driver mutation resulting in asymmetric dimer formation (Fig 7B). Taken together, driver mutations can adopt one of three mechanisms: destabilize the inactive state, stabilize the active state, or both. When viewed on the energy landscape, these can be depicted by the changes in the relative depths of minima, in this case with the outcome of favoring and/or disfavoring the asymmetric or symmetric dimer conformation [264–280].

## The structural basis of oncogenic driver mutations: The KRas example

**Fig 8:**
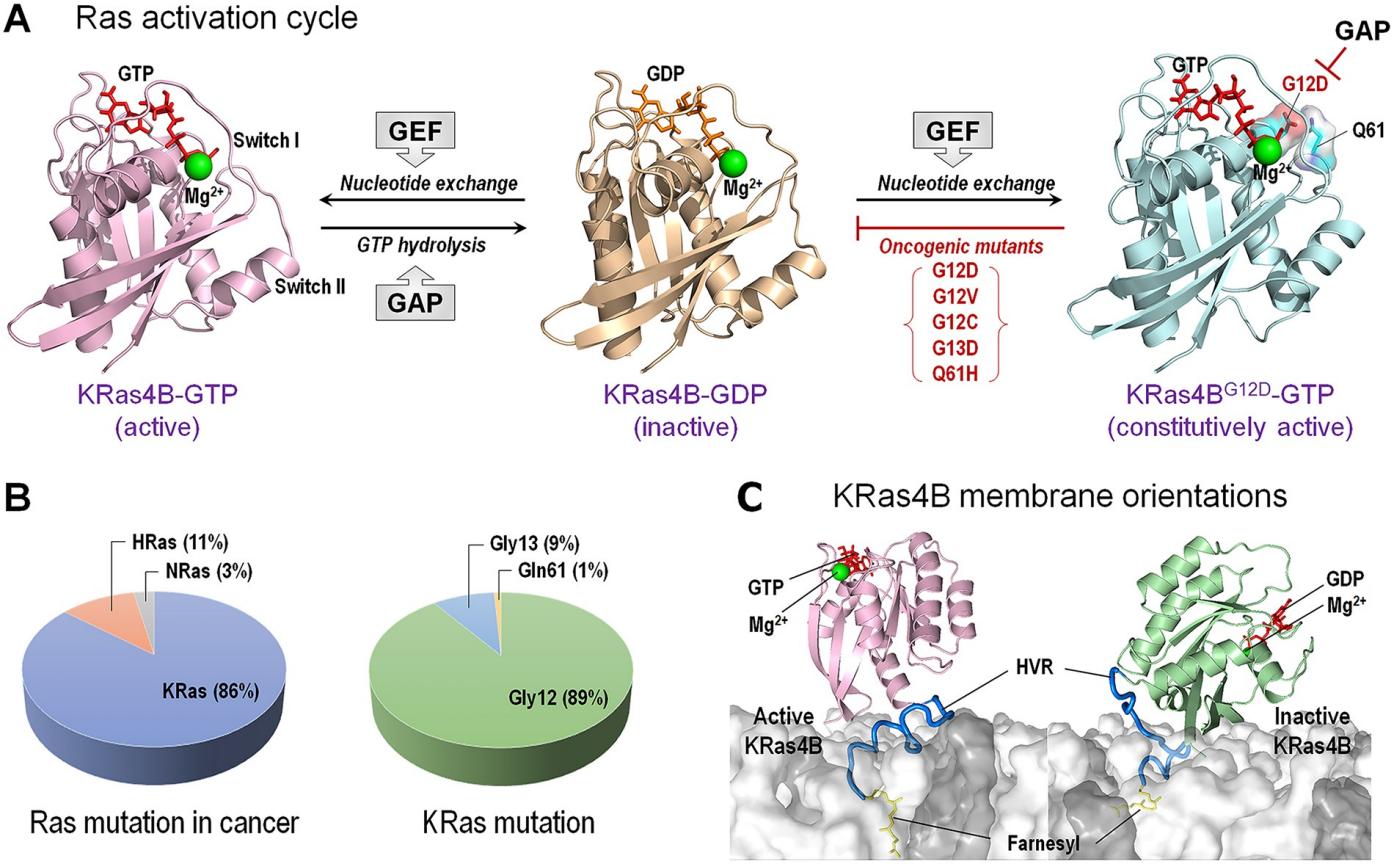
Ras activation and oncogenic mutations. (A) KRas4B is activated by the son of sevenless 1 (SOS1) nucleotide exchange factor (GEF), while GAP inactivates KRas4B via the GTP → GDP hydrolysis. KRas4B oncogenic mutations at the active site Gly12, Gly13, and Gln61 aborts the hydrolysis reaction, keeping the Ras in a constitutively active GTP-bound state. (B) Of those with mutant Ras, KRas is the most highly mutated in cancer. In KRas mutation, Gly12 mutations are the most frequent. (C) The interactions of KRas4B with the anionic membrane composed of DOPC:DOPS (4:1 molar ratio). In the active GTP-bound state, the HVR is in contact with the membrane, but the catalytic domain is away, exposing the effector binding site. In contrast, autoinhibition persists in the inactive GDP-bound state with occluded catalytic domain conformation, yielding the effector binding site is inaccessible. In cartoons, the modeled KRas4B structures were adopted from our simulations [182, 285]. DOPC, 1,2-dioleoyl-sn-glycero-3-phosphocholine; DOPS, 1,2-dioleoyl-sn-glycero-3-phospho-L-serine; GAP, GTPase-activating protein; GEF, Guanine nucleotide exchange factors; HVR, hyper variable region; SOS1, son of sevenless 1.

Like kinases, in the cell, Ras proteins are normally in the inactive state. They are activated following an incoming signal from RTKs. Activation involves exchange of guanosine diphosphate (GDP) by guanosine triphosphate (GTP) by an exchange factor (a GEF) (Fig 8A). GDP-bound Ras proteins are inactive; GTP-bound are active [182, 281, 282]. Ras activation takes place at the membrane [283–285]. Active Ras can activate its effectors; key among these are Raf and PI3K. Their activation stimulates tumor proliferation pathways [189, 286–289]. Unlike kinases, the intrinsic catalytic activity of Ras is very low, orders of magnitude lower than GTPase-activating protein (GAP)-assisted catalysis. Hydrolysis of GTP to GDP, with the help of GAP, switches Ras back to the inactive, resting state. Catalysis requires appropriate positioning and orientation of the respective catalytic residues of Ras and GAP with respect to each other. Oncogenic mutations in Ras suppress catalysis by hindering this coordination. Mutations are common in *RAS*-driven human cancers [114]. The different catalytic scenarios bespeak of the differential modes of action of oncogenic mutations between the kinases and the Ras proteins [182, 290]. Even though in both cases the eventual outcomes are activated signaling, in the case of the Ras proteins, this is by suppressing, not promoting, catalysis. Driver mutations in Ras block the hydrolysis, resulting in constitutive activation. Therefore, unlike in the kinases where a distinct single active conformation with precise distances andorientations among the catalytic groups is achieved by the driver mutations through different mechanisms, in Ras, the mutations lead to multiple states, all of which can suppress catalysis.

Ras has three major isoforms: HRas, Nras, and the splice variants KRas4A and KRas4B. The amino acid sequence of their catalytic domain (residues 1–166) is highly similar, but their hypervariable regions (HVRs) vary [291], as well as their populated conformational states [292]. Hydrophobic PTMs decorate the HVRs and are essential for Ras anchoring in the plasma membrane. Despite the high sequence similarity of the catalytic domains across isoforms, and the fact that the oncogenic mutations are in this domain, their frequency and distribution vary. KRas is the most highly mutated (86%), compared to other isoforms—NRas (11%) and HRas (3%) (Fig 8B); 98% of the mutations are found at the active site Gly12, Gly13, and Gln61, obstructing GTP hydrolysis [114, 293]. KRas Gly12 mutations (89%) are the most frequent, Gly13 (9%), and Gln61 (1%). They are highly mutated in other isoforms as well, including variants, such as G13D (7%) and Q61H (0.6%). Conformational analysis indicated that mutations can change the landscape of proteins [51, 99, 290, 294–306], and this has been shown for HRas, KRas, and NRas [307–311]. Work has especially centered on how key driver mutations, such as those involving substitutions of Gly12, Gly13, and Gln61, abolish GAP-catalyzed hydrolysis [312–315]. In addition, all driver (or other) mutations, wherever they occur, including functional sites as in the case here, have allosteric effects. This has been shown in the case of Q61L in HRas, in which the mutation also allosterically influences the interaction with Raf Ras binding domain (RBD). The timescales (approximately 1 ns) of early molecular dynamics simulations focusing on GAP-mediated hydrolysis were limited, but they did point out that Gln61 of HRas and Arg789 of GAP are vital in GTP hydrolysis [316, 317]. Nonetheless, their brevity limited their ability to observe the influence of GAP binding on Ras conformational transitions. NMR experiments observed that in solution, GppNHp-bound HRas has two interconverting conformations, inactive and active. In a similar vein, simulation of GTP-bound KRas showed that in solution, GTP-bound KRas4B exists in the inactive and active states [182]. The active state of KRas4B in the simulation resembles that of HRas.

Simulations of the GTP-bound wild-type KRas4B complexed with GAP indicated that GAP not only inserts its arginine finger Arg789, aligning the catalytic Gln61 with Arg789 for GTP hydrolysis but also helps stabilize the active state [182]. This arginine finger insertion is thwarted by Ras driver mutations such as those involving Gly12, Gly13, and Gln61 (Fig 8A). In the highly flexible Switch II in the GAP-free state, only a few H-bonds between Gln61 and the GTP γ-phosphate are picked up in the simulations; however, when GAP-bound, the Switch II fluctuations are reduced, permitting formation of the H-bonds. Oncogenic driver mutations disrupt the coordination of the arginine finger Arg789 and the catalytic Gln61, blocking GTP hydrolysis.

The simulations also offer detailed scenarios of how distinct oncogenic mutations differentially suppress catalysis by disturbing the catalytically competent arrangements of Gln61 and Arg789 [182]. Analysis of the snapshots indicates that in the G12C mutant, Arg789 moves away from GTP, preventing the formation of an H-bond between the side chain NE2 atom of Gln61 and the γ-phosphate of GTP; in other mutants, Arg789 still retains its salt-bridge interactions with α- and γ-phosphates of GTP. However, in G12D and G12V, the Gln61 OE1 atom (which extracts hydrogen from the catalytic water) moves away from the γ-phosphorus of GTP; in G13D, the interaction between Gln61 and GTP is lost; in Q61H, His61 cannot coordinate the catalytic water due to distance and/or angle change. Overall, Gly12/Gly13 mutations disturb Gln61 side chain. Mutations can redistribute conformational substates [99, 305, 306], as the KRas simulations of oncogenic mutants also indicate. They can also alter the dynamics. The simulations indicate that G12D, G12V, G13D, and Q61H are more likely to shift the GTP-bound KRas4B ensemble toward the active state than G12C. G12C and G12D trigger larger conformational changes of the GDP-bound state of KRas4B than G12V, G13D, and Q61H. Furthermore, G12C and G12D not only shifted the distribution with large conformational changes but also elicited larger exposure of the nucleotide-binding site, which would facilitate the nucleotide exchange thus activation. Notably, the G12D mutation is the most frequent in KRas4B driven cancers [114] (Fig 8B). Taken together, oncogenic mutations disturb the proper organization of the catalytic residue Gln61 and Arg789 of GAP, impairing GTP hydrolysis, thus retaining Ras in the catalytically active state (Fig 8A). Furthermore, as noted above for HRas Gln61 mutation, even mutations that are directly at functional sites, such as Ras’ Gly12, Gly13, and Gln61 are likely to have additional allosteric effects that would affect their interactions.

Active GTP-bound KRas interacts with the plasma membrane through its farnesylated HVR [285, 318–323]. In vitro and in silico observations suggest that membrane-attached full-length wild-type GDP-bound KRas4B is autoinhibited by its HVR, which is sandwiched between the effector binding site and the membrane [285] (Fig 8C). However, in the GTP-bound state, the interaction weakens, releasing the attachment of the HVR to the catalytic domain. This results in the catalytic domain fluctuating uncontrollably at the membrane with the effector binding surface becoming available. NMR residue chemical shift perturbation analysis and simulations observed that oncogenic mutations shift the landscape toward the exposed effector binding site, with the HVR membrane attached. Therefore, driver mutations can work in multiple ways to both block GTP hydrolysis and ease effector binding.

In resting cells, GDP-bound KRas is inactive, with the equilibrium constant of *k*_off_ (unreleased) significantly larger than the *k*_on_ (released) [324]. In the GTP-bound state, the HVR detaches from the catalytic domain, the farnesyl anchors into the membrane and the catalytic domain can bind its effectors. Oncogenic mutants weaken the HVR interactions with the catalytic domain, increasing the fluctuations of the catalytic domain and thus the population of the exposed site, versus the wild type.

## Low frequency drivers and the concept of “latent driver” mutations

Mutations are classified as being “passengers” or “drivers.” However, biology educated us that it does not pursue a binary “Yes” or “No” game plan. In line with physics and chemistry, conditions matter and changes in the environment will alter the functional outcome. Therefore, the question is then should allosteric mutations be distinguished based on such an unyielding, inflexible, and resolute line?

The free energy landscape suggests that this binary distinction paradigm may not be accurate [40]. As such, driver mutations may be misclassified and not accounted for in cancer treatments [51]. Two issues are at play. For the first, the mutation acts allosterically, except that its prevalence is low. That is, the frequency of the mutation may not surpass a certain threshold to be classified as a driver; however, it acts as a conformational switch, shifting the ensemble of the protein molecule from highly populated inactive to highly populated active state or vice versa. In principle, such mutations can be revealed by NMR or, e.g., through long timescale molecular dynamics simulations. The challenge is which mutation to test out of the many low frequency ones. Detailed analysis of the protein structure may provide clues based on the location, type of residue substitution, and if multiple structures exist, through protein structure comparisons. For the second, the mutation is conformationally “silent”; its frequency is low, and its effect on the ensemble minor. However, should some other cooperative “silent” mutation take place during cancer development, together their effect on the ensemble can be significant. Like driver mutations, together they can turn on (or off) a signaling pathway [247]. Unravelling such “hidden” or “latent driver” mutations among the population of the passenger mutations, and their looming partners, is a compelling aim. Their discovery would extend the standard statistics-based prediction defying the binary Yes/No driver/passenger classification paradigm. As such it can inspire new effective treatments. The free energy landscape theory argues that actionable mutations are not limited to drivers. Even though statistically, and conformationally, a passenger mutation does not correlate with a clinical observation, when coupled with another allosteric effector, such as mutation, pathogen protein, or PTM, it can become actionable. Their underlying mechanisms can be AND (i.e., additive) and graded logic gate integration mechanisms (but not OR) [325].

To understand how an apparent somatic passenger mutation can turn into a driver, we mull over cellular allosteric scenarios [102]. Signaling is often not the outcome of one event; it reflects a combination of events. These can include binding of other protein factors, small molecules, ions, membrane lipids, water molecules, as well as PTMs [233, 326]. A silent mutation can couple with a newly appearing mutation, which is also silent, or with other allosteric events due to changes in the environment in the tumor cell, such as drug or radiation effects. The mutational landscape of cancer is complex, and it is reasonable to expect that among the newly evolved mutations, there will be some that can act cooperatively with existing ones.

**Fig 9:**
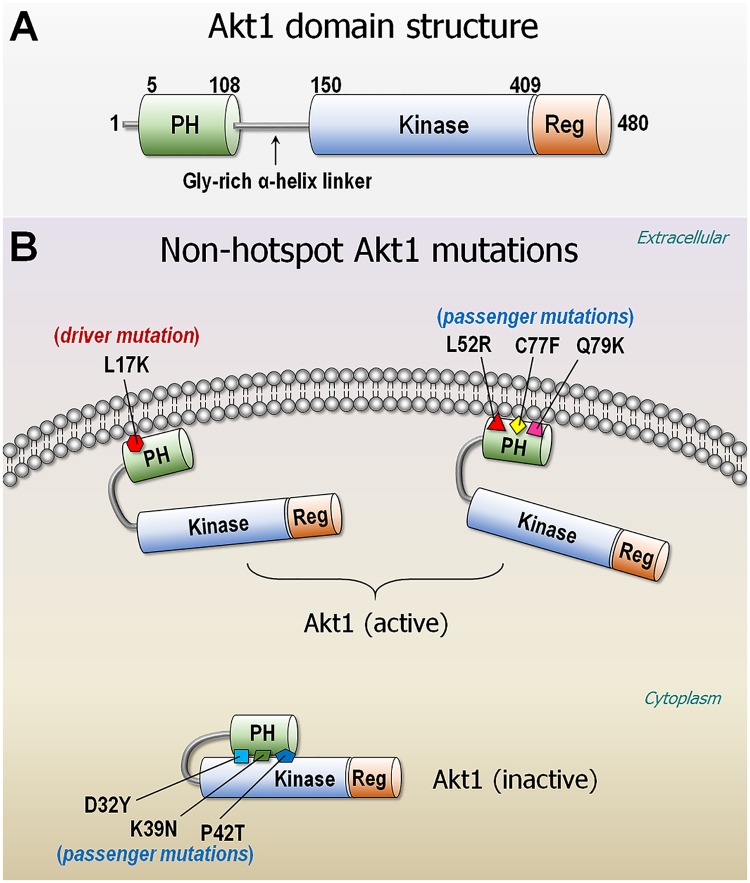
Akt1 domain structure. (A) Akt1 is composed of PH (residues 5–108), kinase domain (residues 150–408), and regulatory domain (residues 409–480). The Gly-rich α-helix linker connects the PH and kinase domains. (B) Non-hotspot, passenger mutations can be functional as the driver mutation E17K. The PH domain mutants, L52R, C77F, and Q79K activate Akt1 as the E17K driver does, whereas D32Y, K39N, and P42T mutants at the interface between the PH and kinase domains reduce Akt1 in the inactive conformation. PH, pleckstrin homology.

Therefore, even though detection of actionable mutations should rest on informatics platforms and clinical trials, fundamental chemical physical considerations of the principles of the behavior of proteins in the cell argue that the annotation should also account for latent drivers that act cooperatively. It is unclear how many patients would benefit from improved identification of actionable mutations incorporating latent drivers; nonetheless, the premise of precision oncology contend that they should not be overlooked. One example is non-hotspot Akt1 mutations that confer constitutive membrane localization acting like the E17K driver mutation [327] (Fig 9), indicating that treatment decisions based only on genetics may overlook vital actionable components. As we discuss below, others include switching pathways in drug resistance. “Latent driver” mutations can also offer an explanation to how impeding an addicted growth pathway can result in rewiring the oncogenic network within short time frames in drug resistance, as well as bear on the question of the rapid evolution of cancers. In both cases, pre-existing, silent, presumably “passenger” mutations partner with newly evolved ones. Identification of a patient’s pre-existing latent drivers can powerfully equip the oncologist with foresight, helping to anticipate drug resistance and decide on drug regimes. The latent drivers’ concept calls for reassessment of genetics-based analysis; however, because their detection requires residue combinations, considerably more genomic sequence data is essential for reliable prediction. Structural data as well as powerful sampling protocols can be invaluable in annotating of these mutations.

That non-hotspot, lower frequency mutations presumed passengers may indeed be functional drivers as illustrated by three examples: (i) The Akt1 pleckstrin homology (PH) domain mutants, L52R, C77F, and Q79K, increased membrane localization of Akt 1 and activated it like the E17K driver did (Fig 9). (ii) In contrast, the PH domain mutants, D32Y, K39N, and P42T, at the interface between the PH and kinase domains abolished the interactions with the membrane, which stabilized the inactive Akt conformation [51, 328]. (iii) The third involved four rare mutations in KRas [329]. Two (K117N and A146T) resemble drivers, one (L19F) presented weakened phenotype, and one (R164Q) resembled wild-type K-Ras. Notably, KRas^D33E^ is also a rare driver mutation [330].

## The conformational basis of targeting in cancer

Exploiting allosteric drugs in precision oncology rests on the premise that even though each driver mutation shifts the ensemble from an inactive to a specific active site conformational state, the conformations of the proteins will vary to a certain extent. This can have two possible consequences: either the location of the allosteric cavity will vary, or more likely, its detailed conformation will differ, resulting in distinct drug preference at the allosteric pocket. This underscores the challenge that precision pharmacology faces in developing allosteric driver mutation-specific drug.

Not all driver mutations are allosteric. Even though no statistics were carried out, it is reasonable to expect that most of the identified, statistically significant driver mutations are not allosteric. These are in the active, or functional site, even though these too will have allosteric effects. Accordingly, drugs can be orthosteric or allosteric. Here our focus is on allosteric drugs. Although the principles of allosteric drugs actions differ from those of orthostric drugs, to date, allosteric drug discovery has largely mimicked orthosteric drug design protocols [331]. Allosteric drugs are more specific; therefore, they are safer. Orthosteric drugs are competitive. They block active sites, turning off protein activity; allosteric drugs act by shifting the population of the active site, impeding its binding to substrates. Allosteric drugs are modulators of function. They can enhance or reduce activity. The structural/chemical difference between allosteric drugs that bind at the same allosteric site and lead to opposing effects can be very small, highlighting the conformational sensitivity in precision oncology [49, 332]. The key determinant of allosteric drugs is the extent of stabilization of the active (or inactive) conformation. This contrasts with orthosteric drugs whose key determinant is affinity [333]. To shift the conformation of the active site, the structures of allosteric drugs include “anchors” and “drivers” [49]. In the allosteric site, the binding of the drug “anchor” atom(s) stabilizes the protein conformation and does not change it as the protein transitions from the inactive state to the active. By contrast, the perturbations caused by the “driver” component “push” or “pull” actions propagate in the protein structure, shifting the conformation to the active (or inactive) state. Allosteric drugs can be noncovalent or covalent. As we discuss below, a recent KRas example epitomizes a specific covalent allosteric action against the G12C driver mutation [116]. Whether noncovalent or covalent, allosteric drugs are uniquely fit to not only target drivers but also latent drivers in precision pharmacology.

The residence times of allosteric drugs in the allosteric pocket, and thus time span of their action, are decided by the drug’s affinity and concentration; however, these do not decide the type of outcome (agonist or antagonist) nor its extent. These are determined by the specific drug—protein interactions [49]. Anchor drug atoms bind to a populated active (or inactive) allosteric site into which their shape and chemistry snugly fit; by contrast, the drug’s driver atoms either sterically collide with protein atoms, in which case they repulsively “push” them, or when attractive, they “pull” them. Anchor atoms stabilize the protein conformation but do not initiate allosteric propagation. On the other hand, the driver atoms’ “pull” or “push” actions lead to allosteric propagation that initiates at these points in the allosteric site and results in a conformational change of the active site. The shape changes it will cause are determined by the distinct push and/or pull actions, which depend on the type of atoms and/or atom groups that execute them.

**Fig 10:**
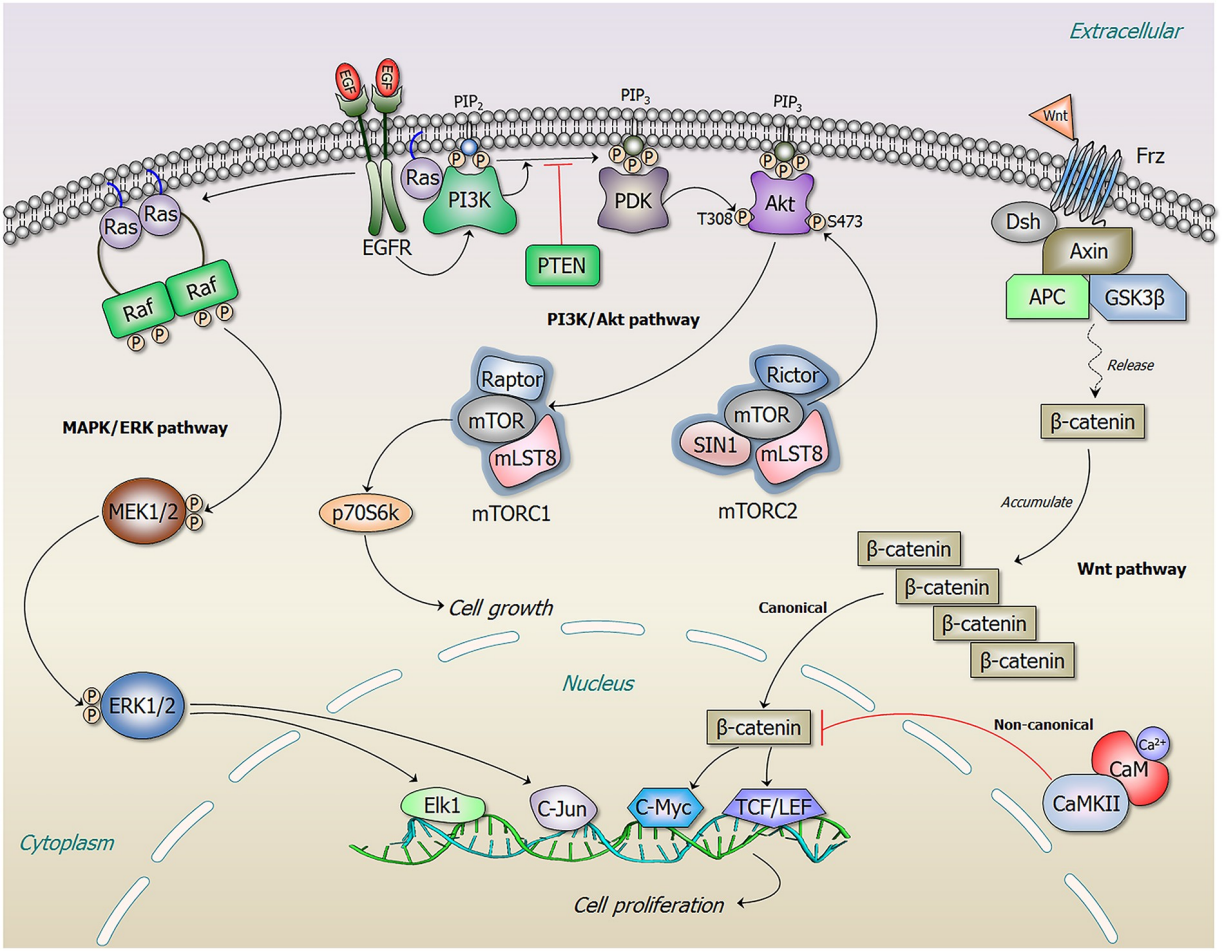
A general scheme of cell-signaling pathway. Representatives shown for the MAPK, PI3K/Akt, and Wnt pathways. In the MAPK pathway, Ras gets activated in the presence of the EGFR signal and forms nanoclusters that promote Raf dimerization. Autophosphorylated, active Raf dimer phosphorylates and activatesMEK 1/2, and subsequently phosphorylates and activates ERK 1/2, leading to cell proliferation. In the PI3K/Akt pathway, both Ras and RTK activate PI3K, recruiting it to the plasma membrane, where PI3K phosphorylates PIP_2_ to produce PIP_3_. The tumor suppressor PTEN can reverse the PIP_3_ production. PIP_3_ recruits both PDK and Akt to the plasma membrane, where PDK and mTORC2 activate Akt, and active Akt phosphorylates the mTORC1, leading to cell growth. The canonical Wnt pathway involves an accumulation of β-catenin in the cytoplasm and translocates β-catenin to the nucleus where it binds to TCF/LEF transcription factors. β-catenin upregulates c-Myc. In the noncanonical Wnt pathway, CaM-bound CaMKII interfere with the canonical β-catenin/TCF/LEF signaling. CaM, Ca^2+^-calmodulin; EGFR, epidermal growth factor receptor; ERK, extracellular signal-regulated kinase; MAPK, Raf/MEK/ERK; MEK, MAPK kinase; mTORC1, mTOR complex 1; mTORC2, mTOR complex 2; PDK, phosphoinositide-dependent protein kinase; PIP_2_, phosphatidylinositol 4,5-bisphosphate; PIP3, phosphatidylinositol 3,4,5-bisphosphate; PTEN, phosphatase and tensin homolog; RTK, receptor tyrosine kinase; TCF/LEF, T-cell factor/lymphoid enhancing factor.

Despite their advantages, allosteric drugs also present challenges [331]. Critical among these are identification of the mutation-specific allosteric cavity [334–349], which is often transient [350, 351] as well as its conformational details, the drug’s driver atoms, and their protein contacts. These will promote the desired allosteric communication. Correct identification is critical, because a misassignment can convert a drug from an agonist to an antagonist. Further, a minor population of the allosteric cavity can imply low affinity, requiring larger drug concentration, thus higher likelihood of toxicity. How to increase drug potency is also unclear. In the case of orthosteric drugs, there are no such conformational hurdles. Experimental structures can provide the active site structures in atomistic detail. These impediments mirror the nature of a drug that aims to tune a conformational ensemble to precisely and potently shift the ensemble toward a specific active site shape. Allosteric drugs exert their action by selecting one—active or inactive—state [49]. An agonist selects an active state, stabilizes it, and increases its population; inverse agonist selects an inactive state, and an antagonist either state. Whether agonist or antagonist, the interactions that anchors form are likely to be unchanged between the active and inactive states, which is not the case for drivers. A minor conformational change at the allosteric site of the inactive conformation by “pushing” a destabilizing collision or an attractive “pulling” of protein atoms can lead to a populated active state with large conformational change. Ras catalytic domain can provide an example. Ras is activated via the exchange of GDP by GTP (Fig 8). It is therefore reasonable that the γ-phosphate acts as the driver. When tested on the GNP-bound versus the GDP-bound Ras crystal structures, two oxygen atoms in the γ-phosphate of GNP were identified as the driver. Their pulling stabilizes the active conformation, with a minor conformational change [49]. By contrast, the crystal structures of protein kinase, phosphoinositide-dependent protein kinase-1 (PDK1) exemplifies a pushing driver. PDK1 is implicated in signaling pathways, which are frequently altered in cancer, such as PI3K andAkt, Ras and MAPK, and Myc (Fig 10), with several identified driver mutations [352].

**Fig 11:**
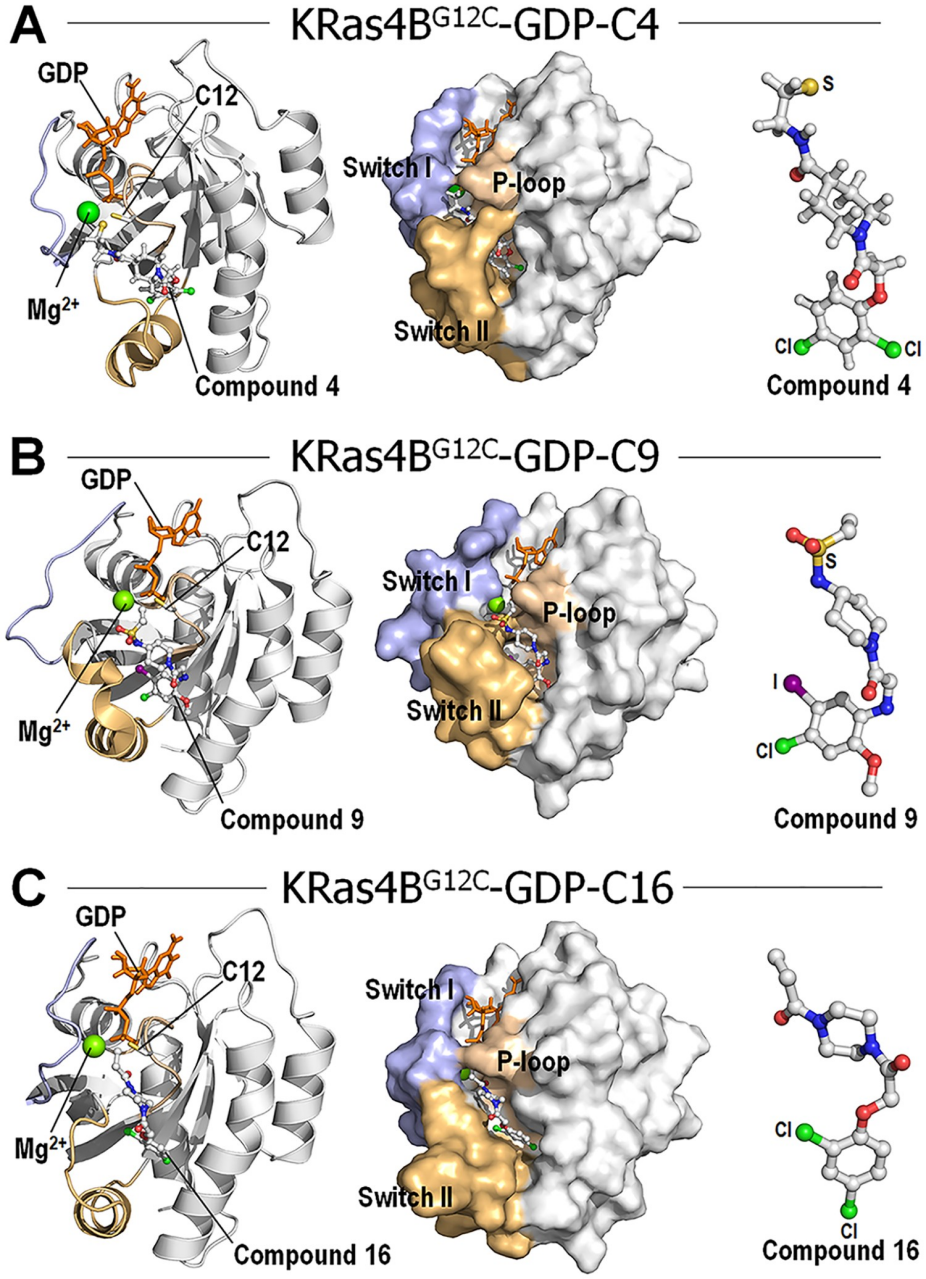
Crystal structures of KRas4B^G12C^ and covalent inhibitors. Cartoon (left panel) and surface (middle panel) representations of the crystal structure of KRas4B^G12C^-GDP in complex with covalently linked inhibitors (right panel) of (A) compound 4, (B) compound 9, and (C) compound 16 (PDB codes: 4LV6, 4LYJ, and 4M22, respectively) [116]. In the protein structures, light green, blue, and orange colors denote the P-loop, Switch I, and Switch II regions, respectively. In the compound structures, C, N, and O atoms are denoted as white, blue, and red spheres, respectively. Hydrogen atom is represented as an edge of stick. Other heavy atoms, S, Cl, and I atoms are marked on the sphere.

Ras also provides an example of covalent, irreversible small molecule drug specifically targeting G12C driver mutation in the GDP but not GTP bound state [333]. Crystallography discovered a small pocket that was not seen in earlier crystal structures, just under the Switch II region (Fig 11). The covalent inhibitors linked to the cysteine, influenced the Switch I and Switch II regions, resulting in the active site favoring GDP rather than GTP, thereby inhibiting Raf’s interaction, thus MAPK signaling [116]. To overcome the challenge in development of covalent inhibitors at flexible sites, covalent docking was compared with empirical electrophile screening for the flexible KRas G12C site, discovering a new, irreversible covalent compound. That compound altered the inactive, GDP-bound state of the G12C mutant, surprisingly accelerating, rather than reducing, SOS-mediated GDP by GTP exchange, thus activation [353]. Further exploration of disulfide tethering of a nonnatural cysteine (KRas^M72C^) detected a new compound (2C07) that binds at the Switch II pocket in the GTP- (or GppNHp) bound state and hampers SOS nucleotide exchange. The attachment of the compound to HRas (at M72C) revealed a new transient cavity. The compound’s binding altered Switch II and by abolishing key polar interactions, drove Switch I further from the GTP. Subsequent design successfully modified the compound such that it could bind both nucleotide states. The ability of the compound to bind to GTP-bound Ras is significant, because in this state Ras activates its effectors, and this is the predominant oncogenic form [354]. This further illustrates the sensitivity of the ensemble, in which a covalent change increases the population times of pre-existing—albeit earlier not captured—pockets near flexible sites.

## Cellular networks

Mutations can inactivate or constitutively activate pathways, as demonstrated for frequent mutations in cancer, such as in *BRAF* and *PIK3CA*, that play casual roles in tumorigenesis. Both orthosteric and allosteric driver mutations and drugs influence cell signaling [103]. Highly oncogenic proteins are often key cellular nodes that link several pathways. Examples include the p53 [355, 356], the Toll-like receptor [357], and Ras proteins [358–361]. Central nodes are hub proteins, which interact with multiple partners [362]. Commonly, interactions take place through shared binding sites [363]. For example, the interactions of Ras with Raf, PI3K, Ral guanine nucleotide dissociation stimulator (RalGDS), Ras association domain-containing protein 5 (RASSF5, also known as NORE1A), and additional effectors are via the same β-sheet surface motif [364]. Multiple factors determine which interaction occurs at any given time, including the effective local concentration, association with, and organization at the plasma membrane, PTMs, and more, which decide and coordinate cell signaling. Wild-type Ras binds and activates Raf, turning on the MAPK pathway; it also binds and activates PI3Kα, turning on the PI3Kα/Akt/mTOR pathway (Fig 10). A KRas molecule binds one effector at a time at a shared site [364]. The affinities of the effectors binding at the same site vary [365]; binding of wild-type Ras to Raf is in the low nanomolar range (approximately 20 nM), whereas it is in the high micromolar for PI3Kα [109, 285, 366, 367] and high nanomolar affinity (approximatley 200 nM) for RASSF5 [368, 369]. If the concentrations of Raf and PI3Kα are comparable, MAPK will take place first, followed by PI3Kα/Akt/mTOR [365]. This order does not only reflect the greater affinity of Ras to Raf but also their differential functions in cell life. The distinct affinities of the effectors at the same Ras surface reflect the different interaction details.

We consider how a driver mutation can alter cell signaling [370–373]. Because the specific residue interactions of the partners with Ras vary, a driver mutation which is at the active site may directly influence partners’ bindings, weakening (or strengthening) one versus the one. Because an effector activates a specific signaling pathway, this can alter signaling and cell fate. If, however, it is an allosteric driver mutation, it will work by shifting the ensemble. The outcome may be a slightly altered surface shape of the active site, which will disfavor binding to an otherwise preferred effector. In a competitive binding scenario as in this case, balanced cell signaling can be disrupted. Therefore, the fallout of a genomic mutation in a protein coding region can quell and trample normal cell signaling through a redistribution of the ensemble.

RASophathy can provide an example of how a covalent linkage of the PTM at specific residue can shift Ras signaling. RASopathies are developmental nononcogenic syndromes commonly caused by germline mutations (rather than somatic mutations found in cancer). They involve dysregulation of the Ras/MAPK pathway. Normal development requires both MAPK and PI3Kα/Akt pathways (Fig 10). In RASopathies, rather than switching between these signaling pathways, the MAPK pathway dominates, stymieing physiological cell cycle regulation. Germline mutations resulting in aberrant ubiquitination are among those documented as associated with RASopathy [374]. Large-scale proteomics [375] identified Ras ubiquitination at lysines 128, 147, and 170 in wild-type HRas. One way through which ubiquitination can work, especially if occurring in the HVR, is impeding membrane anchorage, thus hindering Raf’s activation and MAPK signaling. Therefore, inhibition of ubiquitination would promote MAPK signaling at the expense of PI3Kα/Akt. Furthermore, in line with this, earlier work indicated that inhibiting Ras ubiquitination results in sequestration of Ras on the plasma membrane, promoting its activation, increased levels of extracellular signal-regulated kinase (ERK) 1/2 phosphorylation and lung cancer in cells harboring wild-type Ras [376]. Altogether, this illustrates how alteration of the chemistry of a single residue by relinquishing its charge and adding a bulky group (such as ubiquitin) affect its interaction with the membrane, which can shift signaling to an alternative pathway via effector binding (here Raf rather than PI3Kα) at the same shared site.

The strict definition of precision oncology involves differentiating among distinct driver mutations. A broadened definition may include differentiating among isoforms. HRas, Kras, and NRas have distinct preferred signaling pathways, which is reflected by their patterns of occurrence in specific cancers. Because, however, the sequences of the catalytic domains of the isoforms are highly similar, targeting a specific isoform is challenging. Concomitantly targeting all, as is the practice today, results in high toxicity. This problem is particularly at the forefront for KRas, and especially its KRas4B splice variant, the most highly oncogenic Ras isoform in human cancers. Even though the sequence of the catalytic domains across the isoforms are very similar, the HVR of KRas, but not HRas or NRas, is highly positively charged, populated with several lysines that interact favorably with acidic cell membranes, which is where KRas preferentially locates. Growth factor activation of RTKs triggers many signaling pathways [377] that act in concert in cell proliferation and survival, including the Ras/Raf/MEK/ERK and Ras/PI3K/Akt [249]. Under physiological conditions, all Ras isoforms are activated by stimulated RTK. Oncogenic Ras is activated by its driver mutations, independent of RTK. However, RTK’s role does not end with wild-type Ras activation; it also collaborates with Ras in the activation of PI3Kα. Activation of PI3K requires both binding of PI3K to the C-terminal tyrosine-phosphorylated RTK (e.g., PDGFR) and GTP-bound Ras [378–380]. The Ras binding domain of PI3Kα binds to Ras and gets allosterically activated; however, additionally, RTK’s C-terminal phosphorylated C-terminal motif interacts with the two SH2 domains of the p85 regulatory subunit of PI3Kα, relieving their autoinhibition of the p110 catalytic subunit [381]. In the scenario of oncogenic Ras activating PI3Kα, this interaction is not there; however, uniquely for KRas, phosphorylated Ca^2+^-bound calmodulin (CaM) can act instead. Because CaM is negatively charged, only the HVR of KRas, but not HRas or Nras, can assist in full activation [382, 383]. The resulting quaternary complex of CaM—KRas—PI3Kα can serve as a *KRAS*-specific drug target [384].

## Molecularly targeted therapy, immunotherapy, and gene therapy

Immunotherapy-mediated precision oncology follows different principles and adheres to distinct guidelines [385]. To date, immunotherapy is selective against certain cancers in which the aberrant protein is reachable and can be manipulated by the immune response. Immunomodulatory antibodies targeting T-cell coinhibitory receptors CTLA-4 and PD-1 (programmed death-1) have elicited optimism in several cancers in certain patients, stimulating antitumor immune responses and appearing to offer durable disease control. On the down side, the genomic features and clear specific and robust biomarkers in cancer immunotherapy are still incomplete and challenging to obtain, even though there has been progress in identifying the genomic determinants that can affect the response and resistance of the tumor cells to the treatment. Among the genomic determinants are microsatellite instability, gene copy-number alterations (CNA), and mutations that may affect the immunological response. Here we will not elaborate on this venue of precision medicine, nor on gene therapy, except to note that antibody design is an area our community can consider.

## Conclusions

Translation of personal genomics to precision medicine is fraught with challenges. The underlying complex biology is still lacking, and despite progress, to date, many cell-specific factors are still unknown. Among the advances, are capable and intelligent computer-science—based algorithms to rank molecular alterations that act as drivers of cancer by integrating genomic and transcriptomic data. Such analyses benefit precision oncology, as do the integration of cancer genomics and clinical oncology. They provide comprehensive statistical assessments coupled with phenomenological descriptions. The underlying mechanistic underpinning can amplify them, expanding and substantiating the predictions of potentially actionable mutations, and molecular targets. Here we clarify the physicochemical basis of driver mutations. We relate the free energy landscape of the oncogenic protein to precision oncology, with the hope that this would inspire and dynamize contributions from the chemistry and biophysics communities. Our aim is to articulate a path forward in an area now dominantly populated by other disciplines, which overlook the fundamental underlying behavior of macromolecules in the cell. Together, the life sciences and physicochemical sciences may secure a better future.

Still, challenges abound [51]. Among these are the changes in patterns of methylation epigenetics that influence chromatin reorganization and thereby gene accessibility and silencing, post-transcriptional regulation of signaling molecules by microRNAs, histone modifications, and alternative RNA splicing [386–388]. At least to date, it is not possible to correct aberrant epigenetic marks in a precise, sequence-specific manner. Another obstacle is posed by cell-specific network dynamics, pathway redundancy, and cross-talk, which can be shifted in distinct cell states. Network science and systems-biology—based approaches, e.g., using machine learning and network science principles [2], can integrate multiple data sources and uncover complex changes in a biological system. However, the underlying complex biology is still lacking, and despite progress, to date, many cell-specific factors are still unknown. Furthermore, from our standpoint here, identifying and cataloging driver, passenger, and pre-existing latent driver mutations is enormously difficult. Beyond supervised machine learning to prioritize somatic missense mutations, scores from multiple sequence (protein or DNA) alignments, local amino acid sequence composition, and static physiochemical properties, identifying specific rare mutations that trigger conformational changes that would influence activity is an important mission. Identifying latent driver mutations is particularly challenging. Not only are they rare, at the tail of the distribution curve, but they involve cooperative mutational effects. These may require extensive, long timescale molecular dynamics simulations. NMR experiments can also be helpful in testing predictions.

Cancer develops from genetic alterations, which affect the cell through its signaling network. Cancer cell signaling deregulates the network. Targeted therapeutics aim to block aberrant deregulated protein signaling, and via drug cocktails regimens, redundant pathways that can take over in drug resistance of surviving cancer cells might be stalled [389–405]. Collectively, the basis of actionable mutations and selection of molecular targets should indeed be the informatics definition of driver mutations and precision oncology platforms [24, 406] and the associated clinical trials [158, 407, 408]; however, rare drivers and latent drivers argue that, for higher accuracy, these should be considered as well.

The Pan-Cancer analysis of over 9,000 tumors of 33 different types described the landscape of driver mutations or genes [409]. It indicated an overwhelming presence of nonsynonymous mutations in at least one significantly mutated gene with 2 to 6 mutations per tumor. This further substantiates the vision of precision therapies, which rests on identification and characterization of the drivers [410]. Almost all recurring mutations are highly frequent in different tumor types. To date, the key question is how consistent the correlations between these mutations and therapeutic responses in different cancers are. However, dysregulation mechanisms not considered in the high frequency driver mutation paradigm, such as those in the long tail, may be individually or collectively of high importance too. Classifying them—as well as their combinatorial incidences—may also converge into distinct therapeutic strategies. Our ambition is to reveal these through their underlying conformational mechanisms. The future of precision oncology has been in doubt [411]; we believe that its solid genomic basis, coupled with a grasp of its conformational behavior, will help in better matching the critical genomic alterations with the most beneficial available drug combinations.
