# Correction: Review: Precision medicine and driver mutations: Computational methods, functional assays and conformational principles for interpreting cancer drivers

**DOI:** 10.1371/journal.pcbi.1007114

**Published:** 2019-06-12

**Authors:** Ruth Nussinov, Hyunbum Jang, Chung-Jung Tsai, Feixiong Cheng

Fig 9 is incorrect. The authors have provided a corrected version here.

**Fig 9:**
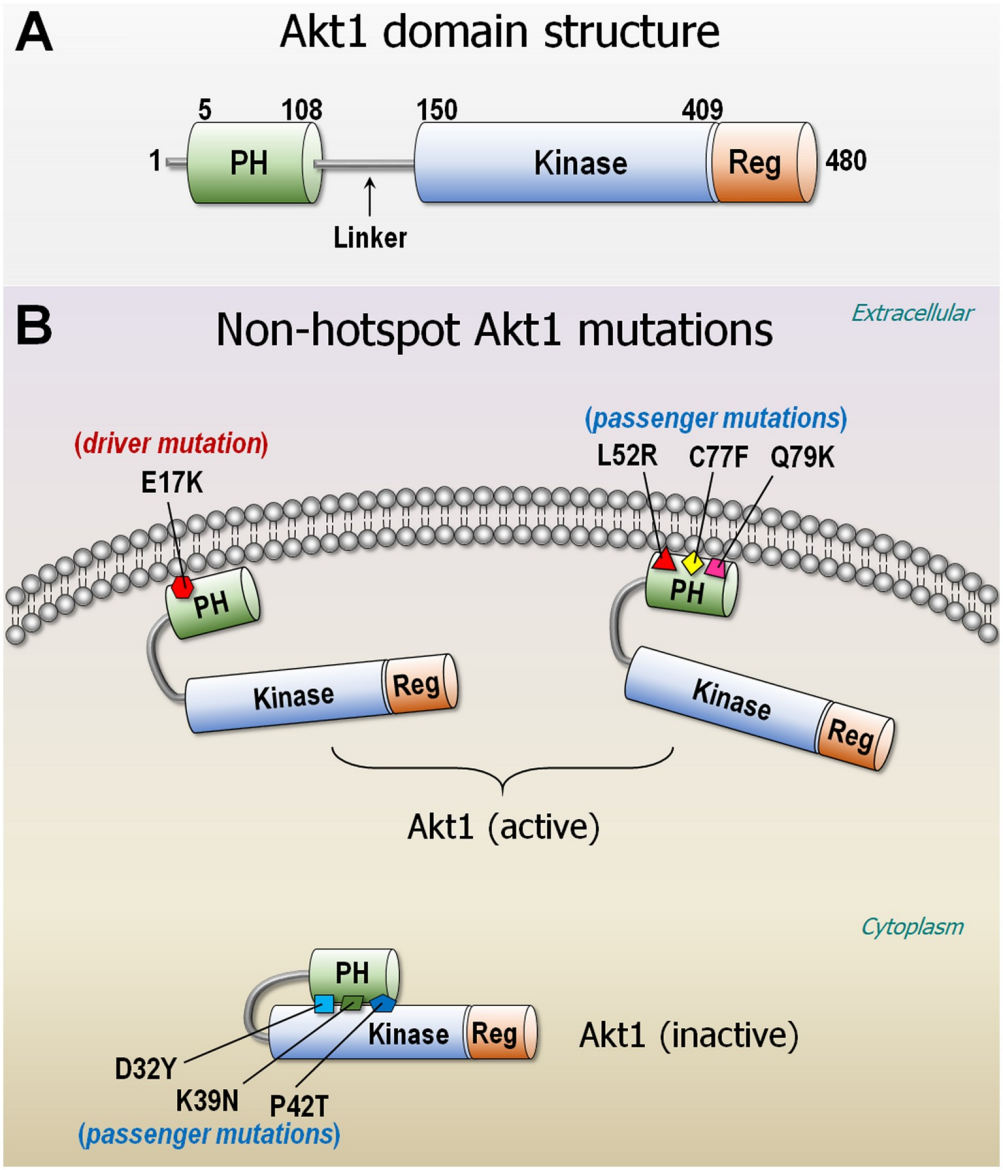
Akt1 domain structure. (A) Akt1 is composed of PH (residues 5–108), kinase domain (residues 150–408), and regulatory domain (residues 409–480). The linker connects the PH and kinase domains. (B) Nonhotspot, passenger mutations can be functional as the driver mutation E17K. The PH domain mutants, L52R, C77F, and Q79K activate Akt1 as the E17K driver does, whereas D32Y, K39N, and P42T mutants at the interface between the PH and kinase domains reduce Akt1 in the inactive conformation. PH, pleckstrin homology.

## References

[1] Nussinov R, Jang H, Tsai C-J, Cheng F (2019) Review: Precision medicine and driver mutations: Computational methods, functional assays and conformational principles for interpreting cancer drivers. PLoS Comput Biol 15(3): e1006658 10.1371/journal.pcbi.1006658 30921324 PMC6438456

